# Communication Tools for End-of-Life Decision-Making in Ambulatory Care Settings: A Systematic Review and Meta-Analysis

**DOI:** 10.1371/journal.pone.0150671

**Published:** 2016-04-27

**Authors:** Simon J. Oczkowski, Han-Oh Chung, Louise Hanvey, Lawrence Mbuagbaw, John J. You

**Affiliations:** 1 Department of Medicine, McMaster University, Hamilton, Ontario, Canada; 2 Canadian Hospice Palliative Care Association, Ottawa, Ontario, Canada; 3 Department of Clinical Epidemiology & Biostatistics, McMaster University, Hamilton, Ontario, Canada; 4 Biostatistics Unit, Father Sean O’Sullivan Research Centre, St Joseph’s Healthcare Hamilton, Hamilton, Ontario, Canada; Boston University School of Medicine, UNITED STATES

## Abstract

**Background:**

Patients with serious illness, and their families, state that better communication and decision-making with healthcare providers is a high priority to improve the quality of end-of-life care. Numerous communication tools to assist patients, family members, and clinicians in end-of-life decision-making have been published, but their effectiveness remains unclear.

**Objectives:**

To determine, amongst adults in ambulatory care settings, the effect of structured communication tools for end-of-life decision-making on completion of advance care planning.

**Methods:**

We searched for relevant randomized controlled trials (RCTs) or non-randomized intervention studies in MEDLINE, EMBASE, CINAHL, ERIC, and the Cochrane Database of Randomized Controlled Trials from database inception until July 2014. Two reviewers independently screened articles for eligibility, extracted data, and assessed risk of bias. Grading of Recommendations Assessment, Development, and Evaluation (GRADE) was used to evaluate the quality of evidence for each of the primary and secondary outcomes.

**Results:**

Sixty-seven studies, including 46 RCTs, were found. The majority evaluated communication tools in older patients (age >50) with no specific medical condition, but many specifically evaluated populations with cancer, lung, heart, neurologic, or renal disease. Most studies compared the use of communication tools against usual care, but several compared the tools to less-intensive advance care planning tools. The use of structured communication tools increased: the frequency of advance care planning discussions/discussions about advance directives (RR 2.31, 95% CI 1.25–4.26, p = 0.007, low quality evidence) and the completion of advance directives (ADs) (RR 1.92, 95% CI 1.43–2.59, p<0.001, low quality evidence); concordance between AD preferences and subsequent medical orders for use or non-use of life supporting treatment (RR 1.19, 95% CI 1.01–1.39, p = 0.028, very low quality evidence, 1 observational study); and concordance between the care desired and care received by patients (RR 1.17, 95% CI 1.05–1.30, p = 0.004, low quality evidence, 2 RCTs).

**Conclusions:**

The use of structured communication tools may increase the frequency of discussions about and completion of advance directives, and concordance between the care desired and the care received by patients. The use of structured communication tools rather than an ad-hoc approach to end-of-life decision-making should be considered, and the selection and implementation of such tools should be tailored to address local needs and context.

**Registration:**

PROSPERO CRD42014012913

## Introduction

With advances in medical care over past decades, end-of-life (EoL) communication and decision-making is now integral to high quality health care, so that patients do not receive unwanted, invasive treatments. Unwanted treatments at the EoL are associated with poorer quality of life and psychological harm for both patients and families [1]. Advance care planning (ACP) and the use of advance directives (ADs) have been proposed as a way of allowing capable patients to record EoL care preferences before a future illness reduces their decision-making ability. However, many patients may not understand their care options, or may not document their care preferences in an AD, with the result that EoL preferences of many hospitalized patients at high risk of death remain unclear to both substitute decision-makers (SDMs) and clinicians, and are often undocumented in the medical record [2].

In order to address these issues, many communication tools (including decision aids, structured meeting plans, and educational interventions) to assist patients and clinicians with EoL decision-making have been developed. There remains uncertainty as to whether the use of such structured communication tools for EoL decision-making in the ambulatory care setting improves the concordance between patient wishes and the actual care received at the EoL. For clinicians and policy-makers, knowing whether these interventions result in improved EOL decision-making and ultimately in better patient outcomes is vital to determine if they are worth implementing in their local setting, given the significant time and resources that may be required to do so. Therefore, we conducted a systematic review to determine the impact of communication tools for EoL decision-making with adult patients in ambulatory care settings on the completion of ADs, the concordance between patient wishes and medical orders for care, and concordance between the care desired and the care received by patients at the EoL.

## Methods

### Protocol and Registration

The protocol for this review is available in the PROSPERO registry at [http://www.crd.york.ac.uk/PROSPERO/display_record.asp?ID=CRD42014012913]

### Eligibility criteria

We included randomized controlled trials (RCTs) or prospective observational studies with a control group (including pre-post post studies in which participants functioned as their own control) published as articles in peer-reviewed journals, restricted to the English language. To be eligible for this review, studies must have included patients over the age of 18, and evaluated a communication tool to assist patients in EoL decision-making, in comparison to a control group. For this study, our definition of “structured communication tool” included traditional decision aids in any format (paper, video, computer, etc.), and other structured approaches to help with decision-making, including organized meeting plans; patient education interventions on EoL care options; or reminders or mailing of ADs. Interventions designed solely for information-sharing (eg. breaking bad news, providing emotional support) were excluded, because although such interventions may affect EoL decision-making, it is not their sole or explicit purpose to do so. Control groups had to receive either use care, a sham intervention, or a minimal/low intensity intervention.

In this paper, we report findings from eligible studies that were conducted in ambulatory care settings. Studies conducted in the inpatient setting, intensive care unit setting, and studies of educational interventions for improving clinicians’ competencies in EoL communication and decision-making will be analyzed and reported separately.

Our primary outcomes were 1) completion of ACP, defined as either completion of an AD or a documented discussion about EoL preferences; 2) concordance between ADs and medical orders for care; and 3) concordance between care desired by patients and the care actually received at EoL. Secondary outcomes included: 1) quality of communication between the patient and family/SDMs; 2) quality of communication between the patient and health care providers (HCPs); 3) patient and family knowledge about EoL care, including options for palliative or intensive care, and knowledge about ADs; 4) health care resource utilization; 5) patient and family satisfaction with EoL care; and (6) for study participants who were exposed to the structured communication tool, the acceptability of the intervention. We subsequently added ‘patient preference for life-sustaining treatments’ as a secondary outcome as it was reported in many studies, and had relevance as a potential surrogate measure of the actual future use or non-use of life-sustaining treatments.

### Information sources and search strategy

We searched the following databases from database inception until July 2014: Medline (1946-July 2014); Embase (1980-July 2014); CINAHL (1982-July 2014); Cochrane Database of Clinical Controlled Trials (2005-July 2014); and ERIC (1966-July 2014). Search terms included: “communication,” “decision-making,” “end-of-life,” “cardiopulmonary resuscitation” (complete electronic search strategies for each database can be found in S1 Table). We also hand searched the reference lists of eligible articles and our personal files to identify further articles for screening.

### Study selection

Retrieved titles and abstracts were screened independently and in duplicate by two reviewers (HC, SO) for potential eligibility using standardized, piloted screening forms. The full text of all articles which passed initial screening by either reviewer were then assessed independently and in duplicate for final eligibility using standardized, piloted eligibility forms. Disagreement about study eligibility was resolved by consulting with a third reviewer (JY). When screening for eligibility, reviewers were not blinded to article authors, journal, or results. Kappa statistics were calculated to assess inter-rater reliability of the screening and eligibility phases [3]. Eligible studies were then divided based on study type into outpatient, inpatient, or intensive care unit settings; or educational interventions for clinicians.

### Data collection process & data items

Study data were collected using standardized, piloted online forms by the two reviewers (HC and SO). Study authors were contacted to clarify study outcomes and methods when they were unclear in the published document. Data collected included study publication information, study dates and population characteristics, study interventions, our primary and secondary outcome measurements, and study methods required to assess the risk of bias in individual studies.

### Risk of bias in individual studies

For RCTs, we assessed risk of bias using the Cochrane risk of bias tool with regard to random sequence generation, allocation concealment, blinding of participants and personnel, incomplete outcome data, and selective reporting [4]. Each domain was assessed independently by both reviewers and reported as being at “high”, “low”, or “uncertain” risk of bias. Studies were considered to be of “low” risk of bias if assessed as being “low” risk of bias in all domains; “uncertain” if uncertain bias in at least one domain, with no domains at high risk of bias; and “high” if there was high risk of bias in any domain. For studies at ‘uncertain’ risk of bias, we attempted to contact study authors to clarify the relevant issue(s), and revised the overall study risk of bias accordingly. Disagreement between reviewers about risk of bias was resolved by consulting with a third reviewer (JY). For observational cohort and case-control studies, we used the Newcastle-Ottawa scale to assess risk of bias [5]. For uncontrolled before-after studies, the National Institutes of Health rating system was applied [6].

### Synthesis of results & sensitivity analysis

We used Revman 5.3 software to conduct our analyses. For each outcome, similar studies were pooled, with a priority given to randomized trials *i*.*e*. data was sought from RCTs first and non-randomized studies were only used in the absence of randomized data. Summarized outcomes (standardized mean difference (SMD) or mean difference (MD) for continuous variables, relative risk (RR) for dichotomous variables) and 95% confidence intervals (95% CI) were calculated using a random-effects model. In our primary analyses, when calculating pooled effect estimates, we restricted to RCTs at low and unclear risk of bias. In sensitivity analyses, we included all studies, including those at high risk of bias, to assess the robustness of our pooled estimates of effect if all studies were included, regardless of risk of bias.

### Investigation of heterogeneity and subgroup analyses

Clinical heterogeneity was assessed by reviewers investigating study populations, interventions, and comparisons. If the studies were considered to be of sufficient similarity for data pooling, study heterogeneity was assessed for each of the outcomes of interest and reported using I^2^ calculations, with values greater than 50% indicating substantial heterogeneity.

### Publication Bias

Publication bias was assessed using visual inspection of funnel plots generated in Revman 5.3, where sufficient numbers of studies existed to permit interpretation [7].

### Rating of Quality of Evidence

We used the Grading of Recommendations Assessment, Development, and Evaluation (GRADE) approach to assess the quality of evidence for each outcome [8]. Outcomes for which the majority of evidence was derived from RCTs was considered to initially be of ‘high’ quality while those from which the majority of evidence was from observational studies started at ‘low’ quality, with both types rated up or down after considering the risk of bias across studies (eg. publication bias); potential biases and their direction within each study; and the imprecision, inconsistency, and indirectness of the evidence. GRADE summary of findings tables were generated using the online GradePRO software [9].

## Results

### Study selection

**Fig 1:**
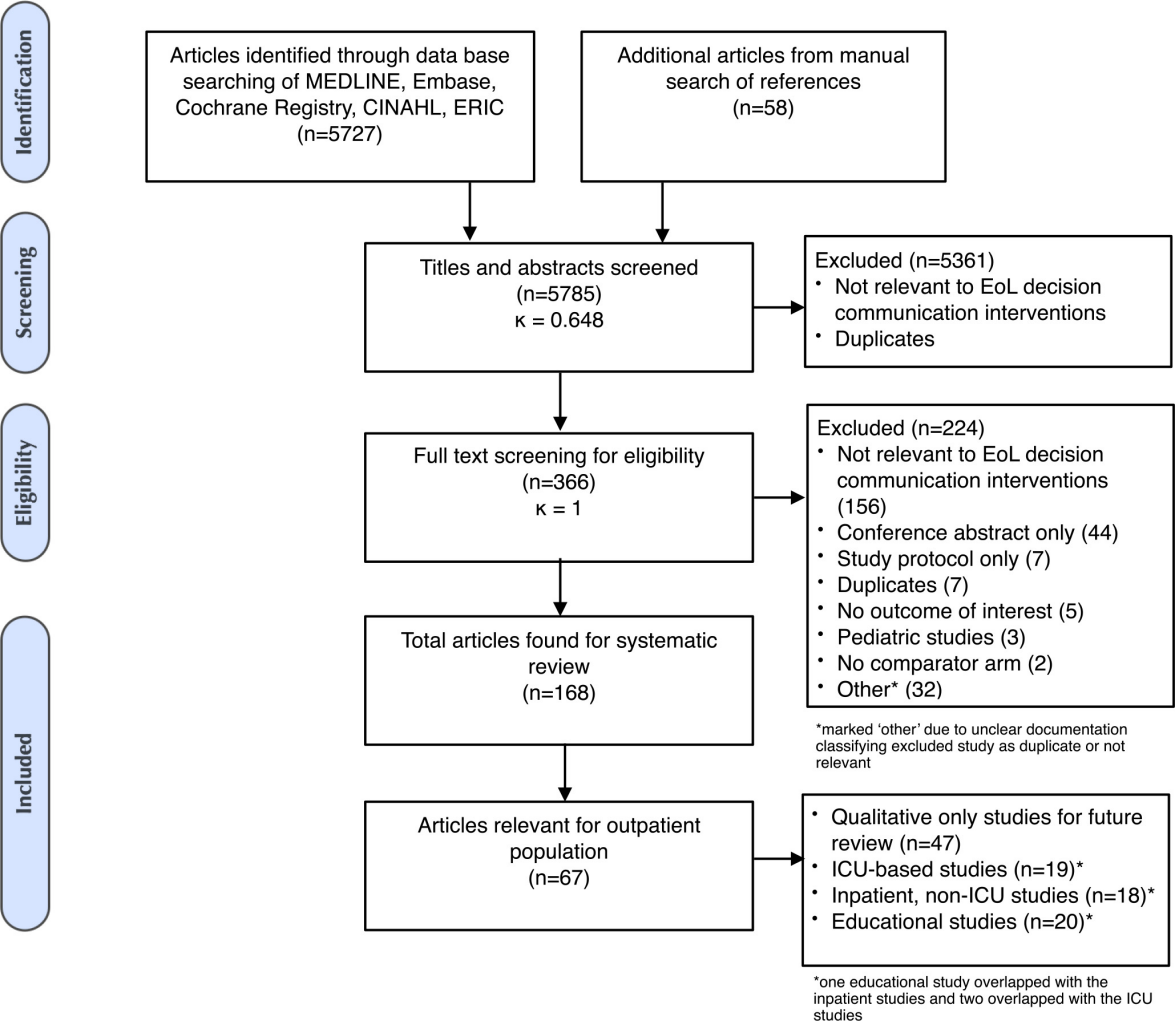
Flowchart of study screening, eligibility, and inclusion.

Initial database searches retrieved 5727 articles. After exclusion of duplicate references and conference abstracts, title and abstract screening resulted in 366 articles selected for full text review (κ = 0.648; 95% CI 0.601–0.695). A total of 121 articles were eligible for our systematic review after full-text review and additional manual reference screening. Of these, 67 reported findings from studies conducted in the outpatient setting and are the subject of this article. (Fig 1)

### Study characteristics

#### Study settings & populations

Studies ranged in publication date from 1991–2014. Sixty-two of the studies were conducted in North America (93%), 2 in Asia (3%), 2 in Europe (3%), and 1 in Australia (1%). Most studies (n = 39, 58%) were of adult participants with no specific medical condition, and the remaining studies focused on participants with cancer (n = 12 studies), cardiac disease (n = 8 studies), renal disease (n = 8 studies), advanced COPD (n = 7 studies), neurologic disease (n = 3 studies), dementia (n = 2 studies), or HIV (n = 2). Some studies included participants with multiple conditions (n = 8). Just under half of studies explicitly included older adults greater than age 50 (n = 29, 43%). A table describing the characteristics in all included studies can be seen in S2 Table.

#### Interventions

Interventions in the eligible studies included verbal discussions alone (n = 9 studies), paper tools alone (n = 9 studies), verbal discussion with paper tool (n = 20 studies), videos (n = 12 studies), computer programs (n = 4 studies), complex multimodal interventions (n = 10 studies), and interventions directed at HCPs rather than patients or SDMs (n = 3). Studies aimed at HCPs were included in this review because either the unit of randomization was the patient (rather than the HCP), or the studies were not educational in nature (eg. chart reminders to discuss ADs with patients).

#### Comparisons

The majority of studies compared the communication tools to usual care (n = 31, 46%), a minimally-intensive EoL intervention (n = 19, 28%), and one compared to a sham intervention (n = 2, 3%). Fifteen studies were uncontrolled before/after studies (n = 15, 22%).

#### Characteristics of excluded studies

Of the 366 studies which underwent full-text review, 224 were excluded. 156 were not relevant to EoL decision-making, 44 were conference abstracts only, 7 were study protocols only, 7 were duplicate articles, 5 did not report any outcomes of interest, 3 were pediatric studies, 2 studies included no comparison arm, and 32 for a combination of reasons, including duplicate or irrelevant studies. A further 101 articles which were purely qualitative in nature, or not based in ambulatory settings, were not included in this analysis and will be reported elsewhere.

### Risk of bias within studies

**Fig 2:**
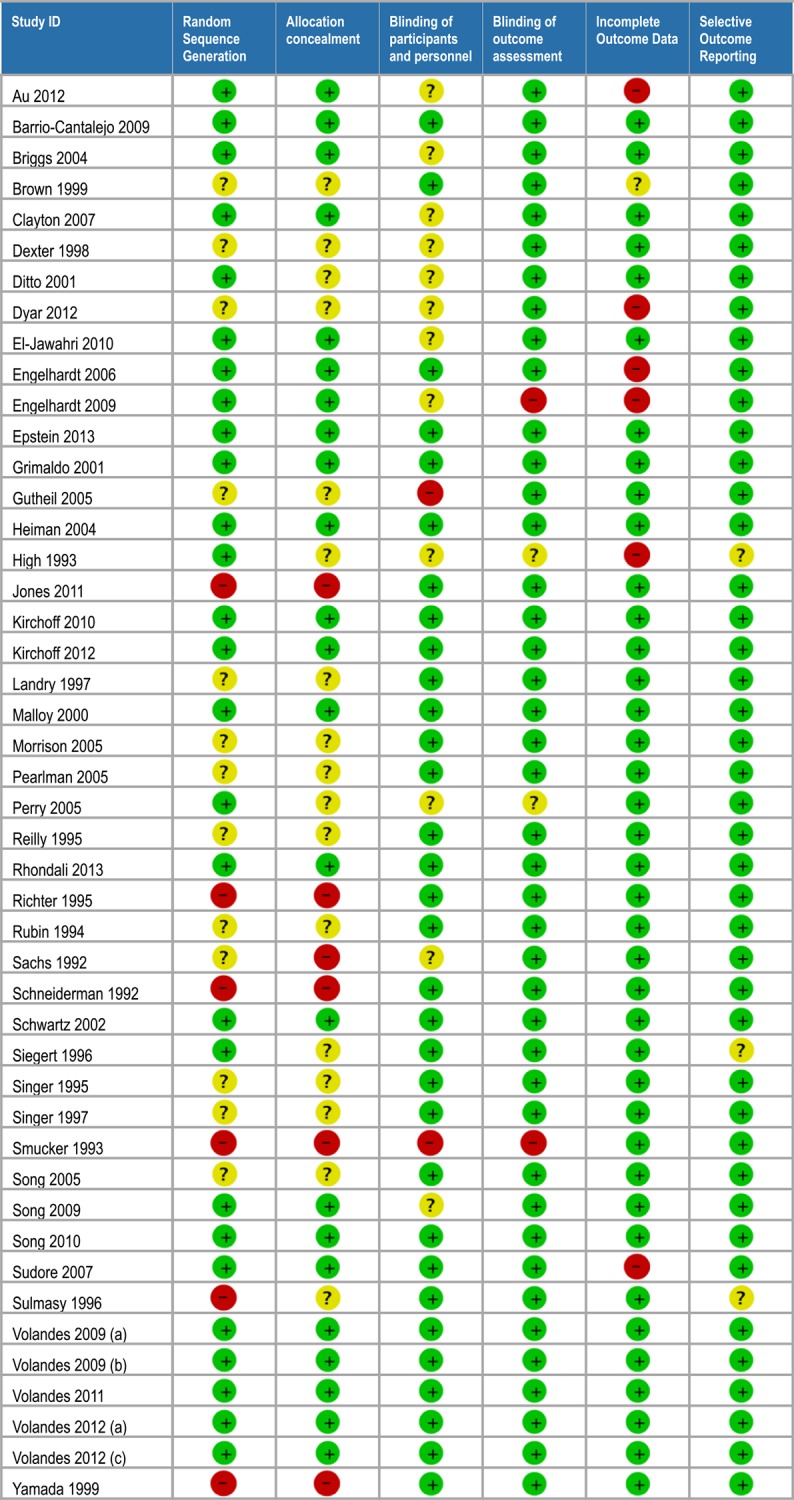
Risk of bias assessments for randomized controlled trials.

Forty-six of the studies were RCTs, of which 12 were considered to be at overall ‘low’ risk of bias [10–21], 15 were considered to be of overall ‘high’ risk of bias [22–36], and 20 of ‘uncertain’ risk of bias despite attempts to contact authors for clarifying information [37–56]. (Fig 2)

**Fig 3:**
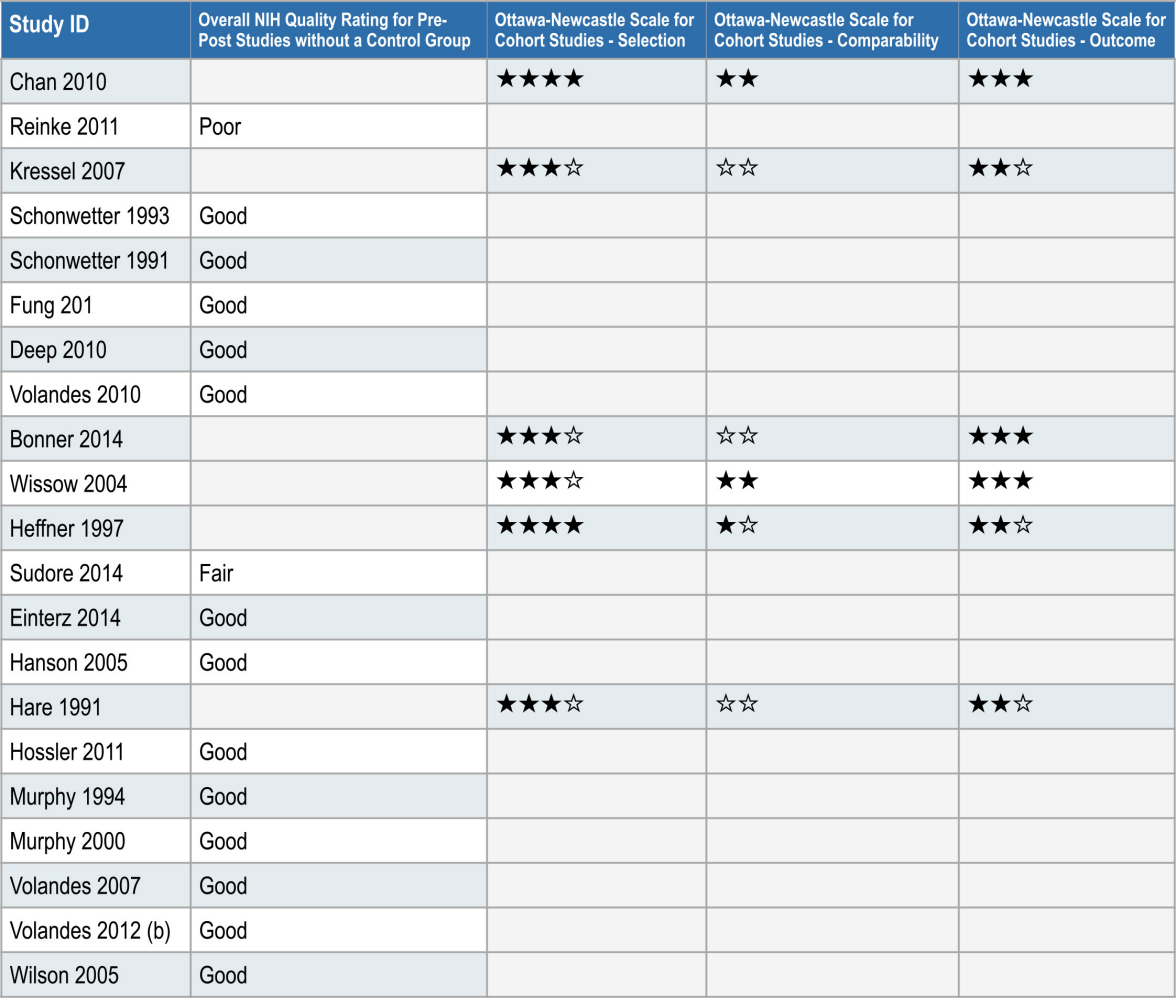
Risk of bias/quality assessments for observational studies.

Of the twenty-one observational studies, 15 were uncontrolled before-after studies, with overall NIH Quality Ratings of “Good” for 13 studies [20,57–68], “Fair” for one study [69], and “Poor” for one study [70]. The remaining observational studies were cohort studies of varying quality, with Newcastle-Ottawa and NIH Quality ratings displayed in Fig 3.

### Primary Outcomes (Table 1)

**Table 1:** GRADE Summary of Findings Table for Primary outcomes.

Outcome	№ of participants (studies)	Relative effect (95% CI)	Anticipated absolute effects in study population (95% CI) *			Quality & Justification
			Without Communication tools for end-of-life decision-making	With Communication tools for end-of-life decision-making	Difference	
**Documented advance directives follow up: mean 1–18 months**	№ of participants: 6030 (12 RCTs)	**RR 1.92** (1.43 to 2.59)	250 per 1000	**472 per 1000** (358 to 619)	**223 more per 1000** (109 more to 370 more)	●●○○ LOW ^1^^ ^^2^
**Documented advance directives discussion**	№ of participants: 1182 (4 RCTs)	**RR 2.31** (1.25 to 4.26)	161 per 1000	**366 per 1000** (226 to 533)	**205 more per 1000** (65 more to 372 more)	●●○○ LOW ^1^^ ^^2^^ ^^3^
**Concordance of patient care wishes with medical orders**	№ of participants: 123 (1 observational study)	**RR 1.18** (1.02 to 1.39)	776 per 1000	**916 per 1000** (792 to 1000)	**140 more per 1000** (16 more to 303 more)	●○○○ VERY LOW ^4^
**Concordance of patient care wishes with received care**	№ of participants: 452 (2 RCTs)	**RR 1.17** (1.05 to 1.30)	490 per 1000	**612 per 1000** (502 to 711)	**122 more per 1000** (12 more to 221 more)	●●○○ LOW ^1^^ ^^4^

CI: Confidence interval; RR: Risk ratio; OR: Odds ratio. High quality: We are very confident that the true effect lies close to that of the estimate of the effect. Moderate quality: We are moderately confident in the effect estimate: The true effect is likely to be close to the estimate of the effect, but there is a possibility that it is substantially different. Low quality: Our confidence in the effect estimate is limited: The true effect may be substantially different from the estimate of the effect. Very low quality: We have very little confidence in the effect estimate: The true effect is likely to be substantially different from the estimate of effect.

* The risk in the intervention group (and its 95% confidence interval) is based on the assumed risk in the comparison group and the relative effect of the intervention (and its 95% CI).

1 Most information used to generate the summary estimate of effect is from studies at 'uncertain' rather than 'low' risk of bias.

2 Large amount of statistical heterogeneity, but between large and small positive treatment effects rather than between positive and negative treatment effects.

3 Most information is from surrogate or variable outcomes rather than from objective and direct outcomes.

4 Insufficient sample to meet optimal information size criteria and 95% CI close to or crosses line of no effect.

Unless otherwise stated, the pooled estimates for primary and secondary outcomes include only RCTs considered to be of ‘low’ or ‘uncertain’ risk of bias. Forest plots including the higher risk studies described in the sensitivity analyses can be seen in S1 Fig

#### 1. Completion of advance care planning (documentation or discussion)

**Fig 4:**
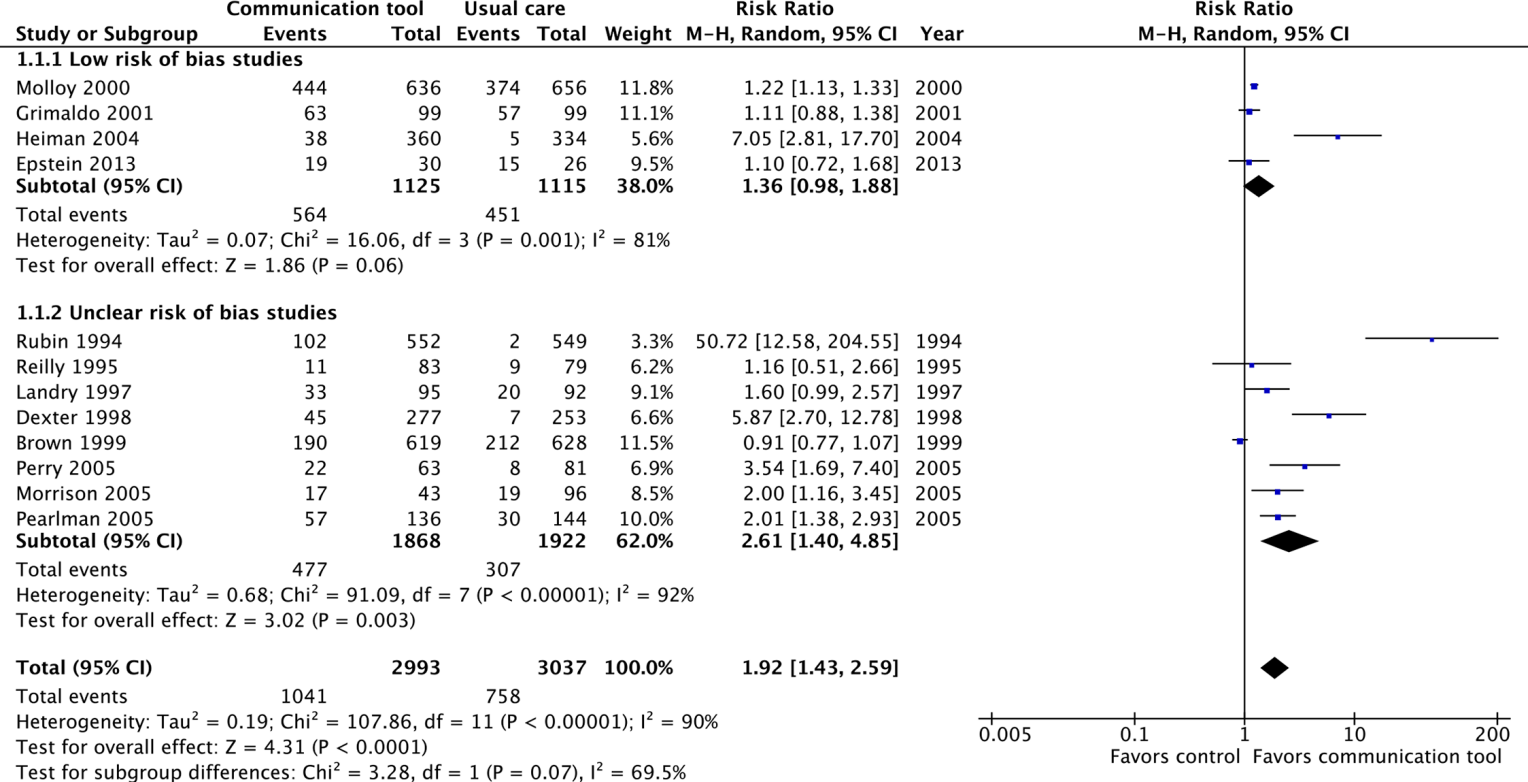
Proportion of patients with documented advance care planning.

Twelve RCTs at ‘low’ or ‘uncertain’ risk of bias reported on the documentation of ADs [11–14,38,40,45–50]. The pooled estimate of effect was statistically significant (RR 1.92, 95% CI = 1.43–2.59, p<0.001, low quality evidence), but with substantial heterogeneity across studies (I2 = 88%). (Fig 4) The pooled estimate was very similar in a sensitivity analysis that included eight ‘high’ risk of bias RCTs which also reported on this outcome [25,26,28,30,31,34–36]. (S1 Fig)

**Fig 5:**
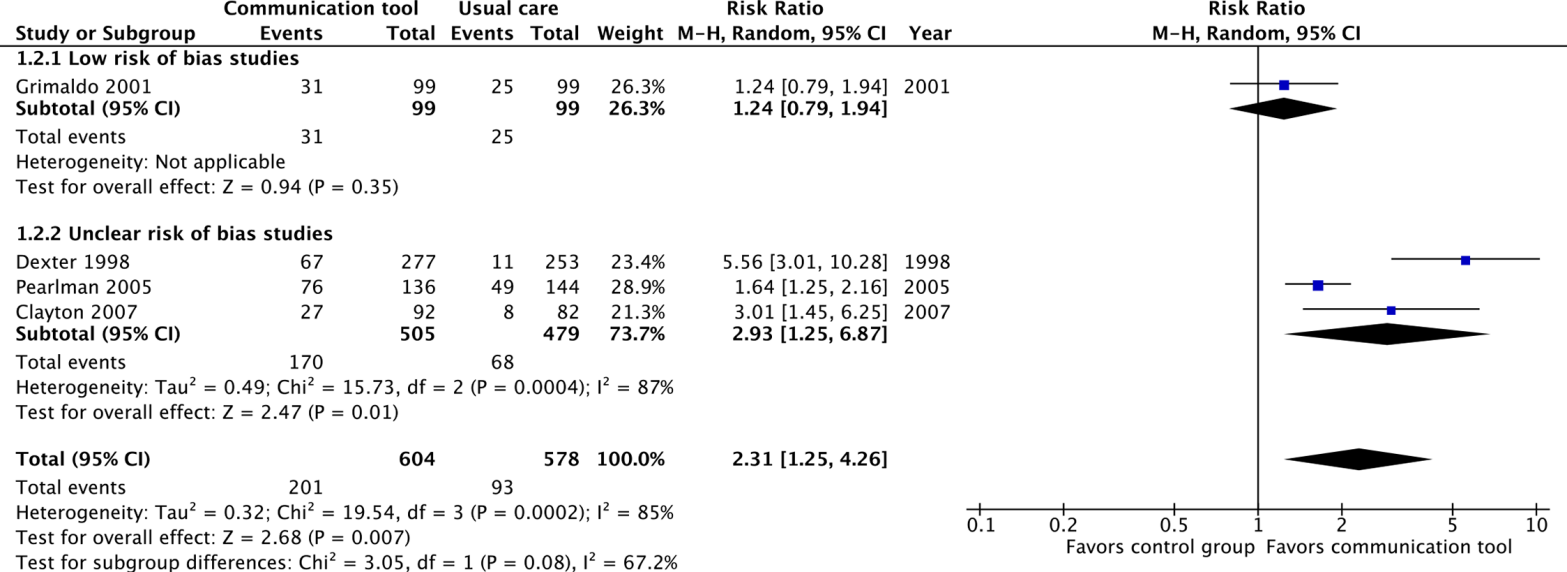
Proportion of patients with documented advance care planning discussions.

Four RCTs at ‘low’ or ‘uncertain’ risk of bias reported on advance care planning discussions [12,39,40,47]. The interventions were associated with a statistically significant increase in the frequency of advance care planning discussions (RR 2.31, 95% CI = 1.25–4.26, p = 0.007, low quality evidence). Again, substantial heterogeneity was observed (I^2^ = 78%). (Fig 5) Sensitivity analysis including four RCTs at ‘high’ risk of bias [13,22,31,35] did not appreciably change these results. (S1 Fig)

#### 2. Concordance between the care desired by the patient and documented orders for medical care

No RCTs reported whether the communication interventions in the outpatient setting improved concordance between patients’ care preferences and documented medical orders for care. A single uncontrolled before-after observational study by Hanson *et al*. of a nursing-home palliative care team with ACP training reported that the use of DNR indicators on the nursing home medical chart increased from 45/58 (77%) to 60/65 (92%) (RR 1.18, 95% CI = 1.02–1.39, p = 0.039, very low quality evidence) amongst patients whose advance directives indicated a preference not to receive CPR [63].

#### 3. Concordance between care desired by patients and care received by patients

**Fig 6:**
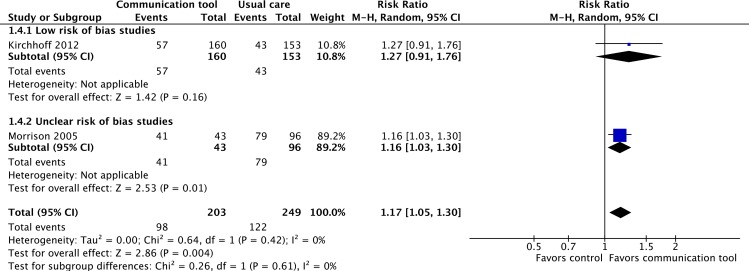
Concordance between care desired by patients and care received by patients at the end-of-life.

Only two studies, one considered to be at ‘low’ risk of bias, the other at ‘unclear’ risk of bias, reported on the concordance between care desired by patients, and the actual care received at the EoL [43,46]. For patients who died, the communication tools were associated with a statistically significant increase in the concordance of care with the care desired by patients (RR 1.17, 95% CI 1.05–1.30, p = 0.004, low quality evidence). No significant heterogeneity was seen (I^2^ = 0%). (Fig 6)

### Secondary outcomes (Table 2)

**Table 2:** GRADE Summary of Findings Table—Secondary outcomes.

Outcome	№ of participants (studies)	Relative effect (95% CI)	Anticipated absolute effects in study population (95% CI) *			Quality & Justification for Ratings
			Without Communication tools for end-of-life decision-making	With Communication tools for end-of-life decision-making	Difference	
**Preference for life-sustaining treatments**	№ of participants: 877 (7 RCTs)	**RR 0.62** (0.41 to 0.94)	283 per 1000	**170 per 1000** (112 to 247)	**113 fewer per 1000** (171 fewer to 36 fewer)	●●●○ MODERATE ^1^
**Concordance of preferred advance directive between patient and substitute decision-maker**	№ of participants: 541 (6 RCTs)	-	The mean concordance of preferred advance directive between patient and substitute decision-maker was **0**	The mean concordance of preferred advance directive between patient and substitute decision-maker in the intervention group was 1.12 standard deviations more (0.62 more to 1.62 more)	SMD **1.12 more** (0.62 more to 1.62 more)	●●○○ LOW ^2^ ^3^
**Quality of communication between patients and SDMs**	№ of participants: 126 (2 RCTs)	-	The mean congruence of preferred advance directive between patient and substitute decision-maker was **0**	The mean quality of communication between patient and SDM in the intervention group was 0.07 standard deviations more (0.28 less to 0.43 more)	SMD **0.07 more** (0.28 less to 0.43 more)	●○○○ VERY LOW ^4^ ^5^ ^6^
**Quality of communication between patients and HCPs**	№ of participants: 126 (2 RCTs)	-	The mean quality of communication between patients and HCP score was **7.65**	The mean quality of communication between patient and SDM in the intervention group was 3.02 standard deviations more (1.26 more to 4.78 more)	MD **3.02 more** (1.26 more to 4.78 more)	●○○○ VERY LOW ^2^ ^5^ ^6^
**Knowledge and literacy for end-of-life care practices**	№ of participants: 427 (4 RCTs)	-	The mean knowledge and literacy for end-of-life care practices was **0**	The mean knowledge and literacy for end-of-life care practices in the intervention group was 0.56 standard deviations more (0.26 more to 0.86 more)	SMD **0.56 more** (0.26 more to 0.86 more)	●●●○ MODERATE ^2^ ^6^
**Knowlege and literacy of advance care planning**	№ of participants: 441 (4 RCTs)	-	The mean knowlege and literacy of advance care planning process was **0**	The mean knowlege and literacy of advance care planning process in the intervention group was 0.3 standard deviations more (0.12 more to 0.49 more)	SMD **0.3 more** (0.12 more to 0.49 more)	●●●○ MODERATE ^2^ ^6^
**Satisfaction with end-of-life care**	№ of participants: 565 (5 RCTs)	-	The mean satisfaction with end-of-life care and care planning was **0**	The mean satisfaction with end-of-life care and care planning in the intervention group was 0.03 standard deviations lower (0.26 lower to 0.21 higher)	SMD **0.03 lower** (0.26 lower to 0.21 higher)	●○○○ VERY LOW ^4^ ^5^ ^6^

CI: Confidence interval; RR: Risk ratio; OR: Odds ratio. High quality: We are very confident that the true effect lies close to that of the estimate of the effect. Moderate quality: We are moderately confident in the effect estimate: The true effect is likely to be close to the estimate of the effect, but there is a possibility that it is substantially different. Low quality: Our confidence in the effect estimate is limited: The true effect may be substantially different from the estimate of the effect. Very low quality: We have very little confidence in the effect estimate: The true effect is likely to be substantially different from the estimate of effect.

* The risk in the intervention group (and its 95% confidence interval) is based on the assumed risk in the comparison group and the relative effect of the intervention (and its 95% CI).

1 Suspected publication bias based upon visual inspection of funnel plot.

2 Most information is from studies at 'uncertain' rather than 'low' risk of bias.

3 Large amount of statistical heterogeneity, but between large and small positive treatment effects rather than between positive and negative treatment effects.

4 Most information is from studies at high risk of bias.

5 Most information is from surrogate or variable outcomes rather than from objective and direct outcomes.

6 Insufficient sample to meet optimal information size criteria and 95% CI close to or crosses line of no effect.

#### 1. Patient preferences for life-prolonging treatments

**Fig 7:**
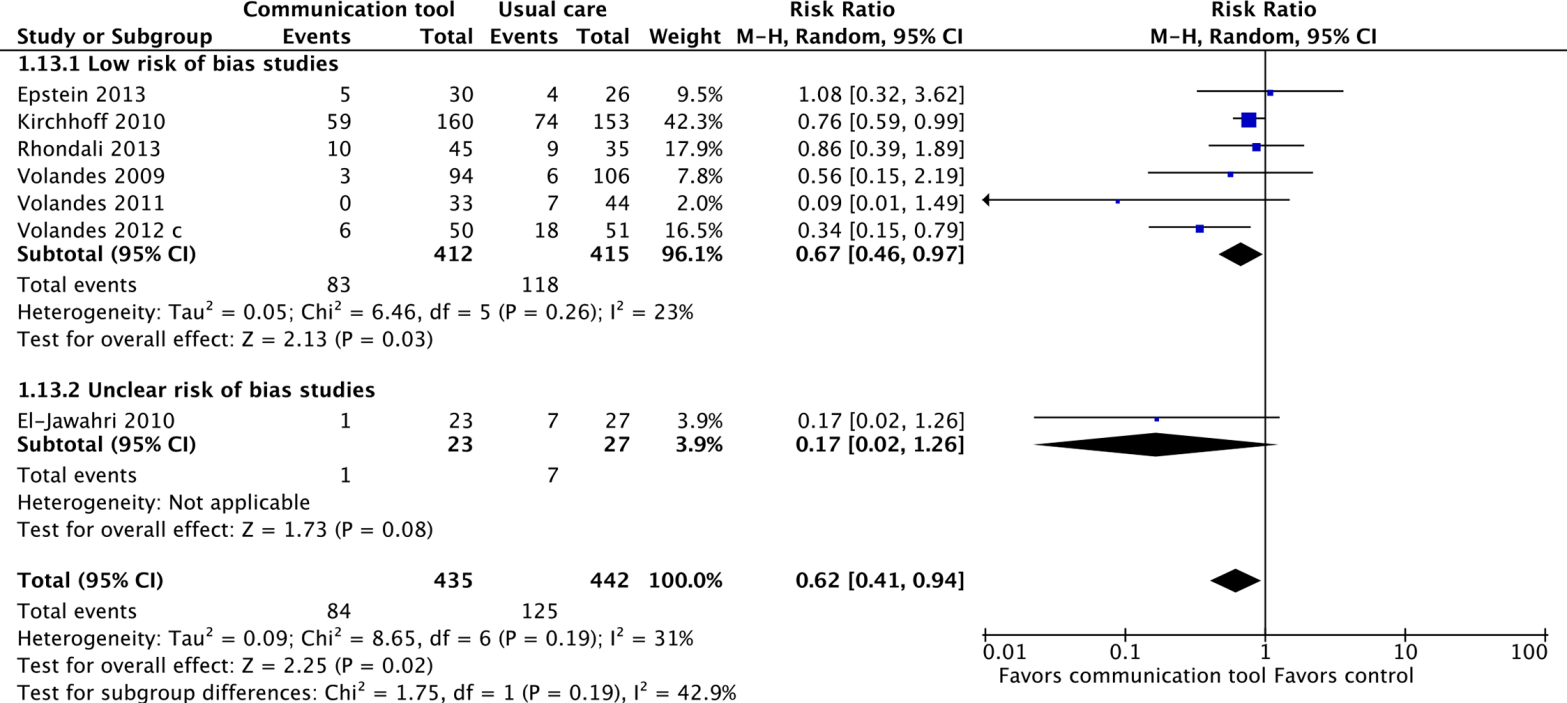
Patient preferences for life-prolonging as opposed to comfort care.

**Fig 8:**
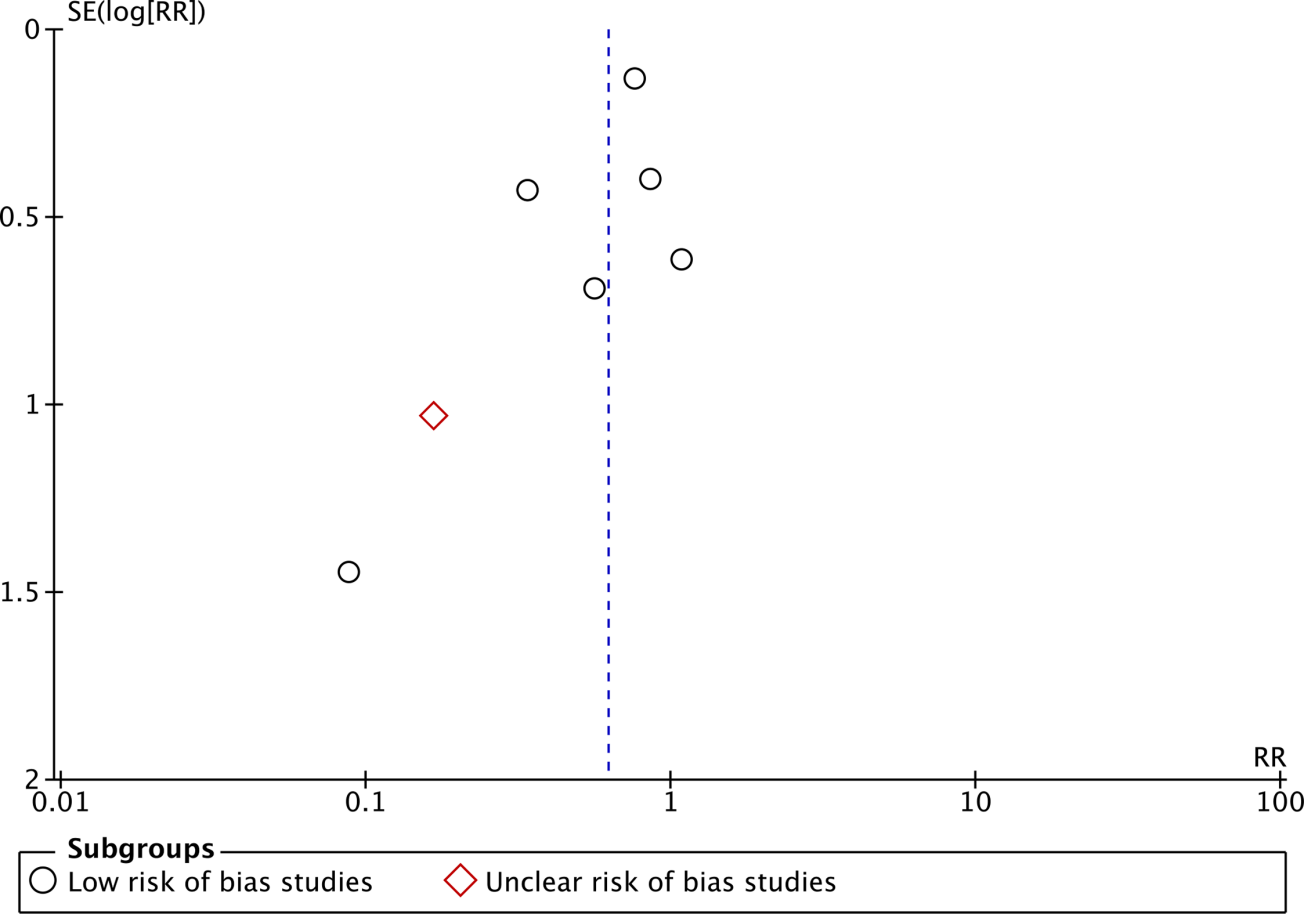
Funnel plot for outcome “Patient preferences for life-prolonging treatments”.

Seven RCTs considered to be of ‘low’ or ‘uncertain’ risk of bias reported patient preferences for life-supporting treatments [11,15,17,19,21,42,43], and found that communication tools reduced patients’ stated desire for life-supporting treatment (RR 0.62, 95% CI = 0.41–0.94, p = 0.02; I^2^ = 2%). (Fig 7) For this outcome, publication bias was suspected based upon visual inspection of the funnel plot. (Fig 8) In a sensitivity analysis that included three ‘high’ risk of bias trials, the magnitude of effect was smaller but still statistically significant [25,32,36]. (S1 Fig)

#### 2. Quality of communication between the patient and family/SDM

Measurement of quality of communication between patients and families/SDMs was reported in two ways in the included studies: some reported concordance between patients and SDMs about preferences for EoL care while others used rating scales to assess the quality of communication.

**Fig 9:**
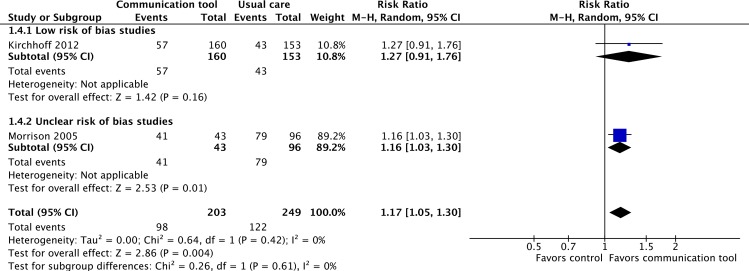
Quality of communication between patient and substitute decision-maker (SDM)—concordance between patient and SDM for level of care desired at end-of-life.

Six RCTs of ‘low’ or ‘uncertain’ risk of bias reported concordance between patients and SDMs for level of EoL care desired [10,16,37,47,51,55], demonstrating a statistically significant improvement in concordance scores (SMD 1.12, 95% CI = 0.62–1.62, p<0.001). Substantial heterogeneity was noted (I^2^ = 81%). (Fig 9) None of the eligible studies evaluating this outcome were judged to be at high risk of bias.

Two RCTs, both considered to be of ‘high’ risk of bias reported scores assessing the quality of communication between patients and SDM [27,29]. Neither study found a statistically significant difference between intervention and control groups for quality of communication between patients and SDMs.

#### 3. Quality of communication between the patient and HCPs

**Fig 10:**
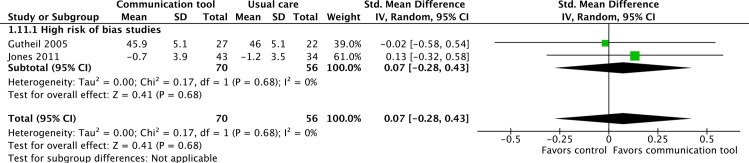
Quality of communication score between patients and health care providers.

Only two RCTs of ‘low’ or ‘uncertain’ risk of bias reported quality of communication scores for patients and HCPs [16,56], both using the same quality of communication score. In both, the intervention was associated with a statistically significant improvement in the quality of communication score (MD 3.02, 95% CI = 1.26–4.78, P<0.001; I^2^ = 51%). (Fig 10) When two ‘high’ risk of bias studies were included in the sensitivity analysis, the magnitude of effect decreased but remained statistically significant [22,29](SMD 0.55, 95% CI = 0.01–1.09, p = 0.04). (S1 Fig)

#### 4. Patient and family knowledge related to advance care planning

**Fig 11:**
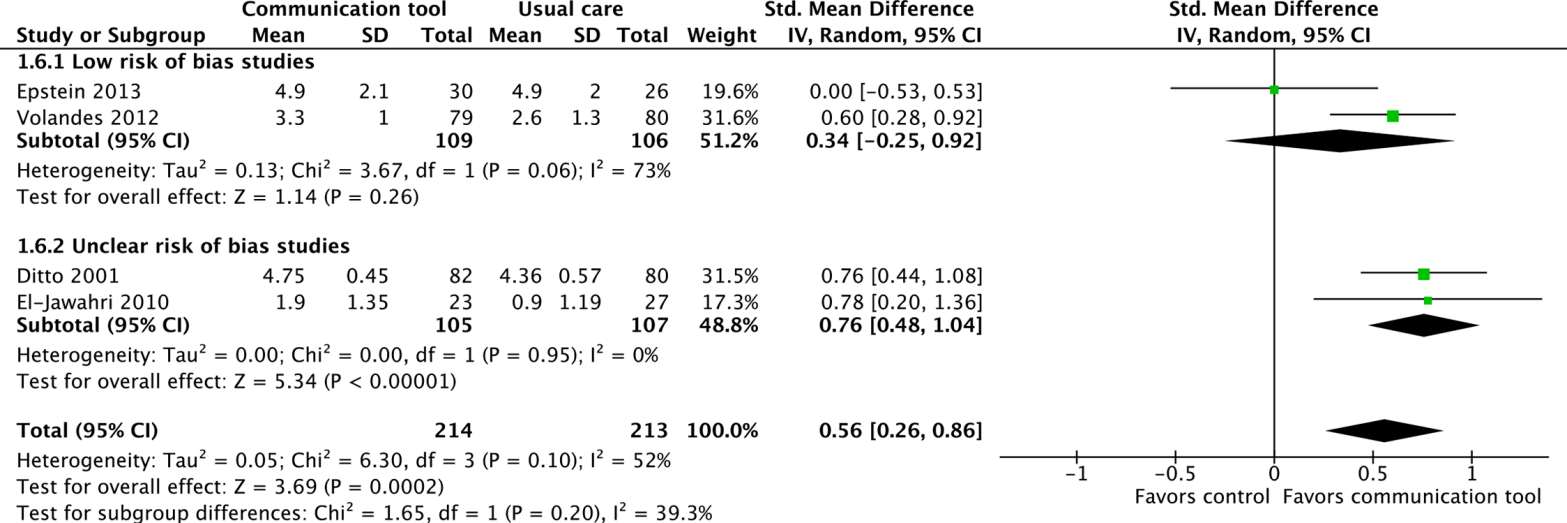
Patient and family knowledge of life-supporting treatments.

Four RCTs of ‘low’ or ‘uncertain’ risk of bias reported upon patient and family knowledge of life supporting treatments, such as CPR or mechanical ventilation [11,21,41,42], with a statistically significant improvement in knowledge scores (SMD 0.56, 95% CI = 0.26–0.86, p<0.001; I^2^ = 52%). (Fig 11) Two studies considered to be of ‘high’ risk of bias were also found, and their inclusion in a sensitivity analysis did not appreciably change the estimate of effect [24,34]. (S1 Fig)

**Fig 12:**
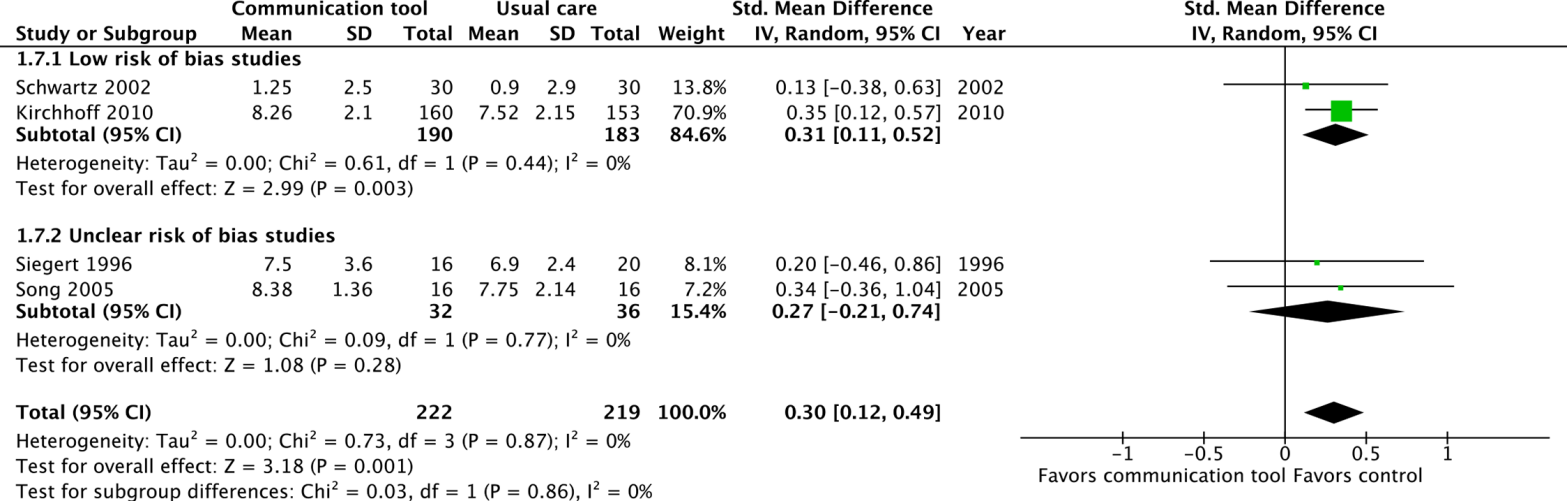
Patient and family knowledge of advance care planning.

Four separate RCTs also evaluated the effects of communication tools on patient and family knowledge of advance care planning, two at ‘low’ risk of bias [43,51] and two of ‘uncertain’ risk of bias [52,55]. A statistically significant improvement in knowledge scores was seen (SMD = 0.30, 95% CI = 0.12–0.49, p = 0.001: I^2^ = 0%), with no change in a sensitivity analysis when one high risk of bias study was included [27]. (Fig 12, S1 Fig)

#### 5. Patient and family satisfaction with EoL care

Only one RCT considered to be of ‘low’ risk of bias [39] reported patient and family satisfaction with EoL care, finding no differences with the use of communication tools. Four RCTs considered to be of ‘high’ risk of bias [24,26,27,29] also reported measures of patient and family satisfaction with EoL care, and likewise did not find any significant improvement or worsening in patient or family satisfaction with EoL care.

#### 6. Health care resource utilization

There were two RCTs at low risk of bias that reported health care utilization, one finding a non-statistically significant reduction in the total number of ICU admissions between the intervention and control groups (0 (0%) v. 3 (12%), p = 0.093) [11], and the other finding a reduction in the number of hospitalizations (143 [27%] v. 290 [48%], p = 0.001) and total health care costs ($3490 USD [SD 1499] v. $5239 USD [SD 1812], p = 0.013).

Three RCTs considered to be of ‘high’ risk of bias also reported health care resource utilization, one finding no statistically significant difference in health care costs ($12,123 USD v. $16,295 USD, p = 0.18) [26], one finding significant reductions in ER visits (3.69 [SD 6.14] v. 5.35 [SD 12.87], p = 0.001) and inpatient admissions (2.44 [SD 5.11] v. 4.33 [SD 16.26], p<0.05) at 4 months [25], and one finding no difference in the mean ICU days per patient (2.5 [SE 1] v. 3.1 [SE 1], p>0.2) or mean hospital days per patient (40.8 [SE 4.4] v. 33.1 [SE 3.6], p = 0.18) [32].

No pooled results were generated due to the heterogeneity of outcomes reported. The overall quality of evidence for resource utilization was considered to be ‘low’ given the low quality of studies and the inconsistency of the effects in the few trials which reported measures of resource use.

#### 7. Patient and family acceptability of the intervention

Nine RCTs [11,15,21,34,38,39,42,53,54] reported on patient and/or family acceptability of the EoL communication tool studied. In all studies, the majority of participants found the communication tools acceptable. All studies which compared acceptability of the tools to usual care found them to be equally or more acceptable than usual care [11,15,34,53,54].

## Discussion

Our systematic review identified 46 RCTs and 21 observational studies which assessed the effects of structured communication tools on EoL decision-making in a wide variety of adult outpatient populations. Although many of the studies addressed populations with specific comorbidities, the majority of studies addressed populations with no specific medical condition. We found low quality evidence that structured communication tools to assist with EOL decision making in ambulatory care settings may increase the completion of ACP (discussions or AD documentation), very low quality evidence (one non-randomized before-after study) that they may increase concordance of patient preferences with medical orders for the use/non-use of life sustaining treatments, and low quality evidence that they may increase concordance between care desired and care received at EOL (Table 1).

It is unknown whether this is achieved by i) increased translation of AD documents into the acute care setting; ii) improved patient and/or SDM knowledge of the limits of acute care, resulting in more realistic expectations for aggressive care; iii) improved verbal communication between patients, SDMs, and health care providers; or a combination of the above factors. Our secondary outcomes provide support for the latter two mechanisms, with moderate quality evidence suggesting that communication tools result in improved health literacy for patients and SDMs, and reduce the proportion of patients with preferences for life-prolonging care. Very low quality evidence suggests that the use of structured communication tools improves quality of communication between patients, SDMs, and health care providers (Table 2).

Our review found only very low quality evidence about other ‘downstream’ outcomes of ACP—namely patient satisfaction with EoL care and health care resource utilization. Only a small number of studies of limited quality reported the long-term effects of the communication tools on these important outcomes, with no major effects seen on patient satisfaction, and variable results upon resource use. The lack of evidence for the effectiveness for the interventions upon these outcomes may be due in part to their use in the ambulatory setting—we would anticipate that a very large sample size and prolonged follow-up period would be required to find a significant effects from what is, in effect, a ‘preventative’ treatment aimed at avoiding potentially unwanted, invasive care in the future.

We believe that eliciting patient wishes for EoL care planning is an inherently valuable practice. Our review provides evidence (albeit of low quality) that the use of such tools have a “class effect” and may increase completion of ACP (discussions or documentation of ADs). The wide variety of populations and interventions studied in the articles we reviewed make it difficult to identify a single ‘best’ tool to adopt, especially considering not all of the interventions we studied had large or positive effects. It seems reasonable that implementation of tools should be tailored to the local context (disease population, severity of illness, *etc*.). Given the low confidence in the effect of these tools on ACP completion and other related outcomes, we suggest that those who choose to implement structured EOL communication tools in clinical practice track performance related to outcome(s) of interest, such as completion of ACP. By doing so, end-users can ensure that these resources are having the desired effect.

Finally, one of the difficulties in our review was the limited reporting of important clinical outcomes. We would support a standardization of outcomes for studies of advance care planning. A structured approach to ACP outcomes should include measures of knowledge (patient and SDM understanding of ACP), process (increased completion of ADs, improved communication between patients, SDMs, and HCPs) and outcome (concordance between care desired and received; patient satisfaction; and resource use). These outcomes should also be considered at the patient level (*eg*. patient preferences), SDM level (*eg*. concordance of SDM with patient preferences), and system level (*eg*. concordance between care desired and care received). Future research should focus on simple interventions, and study their use across a wide variety of patient populations. As the documentation for ACP can vary from region to region, and change over time, the interventions should ideally focus on eliciting values and preferences for EoL care in general, rather than focus on completing the specific document used in the investigator’s region.

### Strengths

The strength of our study lies in its rigorous search strategies; the use of two separate authors in assessing studies for screening, eligibility, and risk of bias, with secondary checking and verification of data extraction; as well as in our use of GRADE to assess the overall quality of evidence for each outcome. Our study also explicitly included a wide variety of interventions to assist in EoL decision-making, including traditional decision aids, structured meetings, and educational interventions, allowing us to review the full spectrum of tools which have been published in peer-reviewed journals, some of which may not have been traditionally identified as a ‘decision-aid.’

### Limitations

Our study has two major limitations, one related to our methods, and the second related to the studies we found. Firstly, identifying studies of interventions to facilitate EoL decision-making is challenging due to a lack of consistent terminology for such interventions. In many cases, only by carefully reviewing a study’s methods and outcomes could it be determined that the intervention’s purpose was to facilitate EoL decision-making. Evidence of this difficulty is seen in the large number of studies found by hand-searching in addition to our computerized literature searches. Given these difficulties, it is possible that our review failed to identify some potentially relevant articles, despite our rigorous search.

Secondly, our review is limited by the highly heterogenous nature of the populations and interventions studied. Across studies, ‘usual care’ varied between no intervention and complex encouragement of ADs, depending on local practice. Despite the wide variety of populations and interventions, the studies generally revealed either positive or neutral effects for all of our outcomes of interest, suggesting that the use of a structured communication tool has a “class effect” and is overall more likely to lead to improved outcomes compared to less-structured approaches used during usual care.

## Conclusions

A wide variety of communication tools for EoL decision-making have been evaluated in many outpatient populations. Overall, the available evidence suggests that structured communication tools to assist in end-of-life decision-making may improve communication processes and some downstream patient level outcomes, but uncertainty about the true magnitude of effect remains because of the low quality of the existing evidence. While awaiting more rigorous evaluation, use of structured communication tools, rather than ad-hoc discussions about the end-of-life, should be considered. Given the heterogeneity of populations, interventions, and effects, more work is needed to guide the selection, adaptation, and tailored implementation of such tools to local care settings and contexts. Effectiveness of implementation efforts needs to be monitored to assess the success of such interventions.

## Supporting Information

S1 Fig Sensitivity analyses. (PDF)

S1 Table Electronic search strategies. (PDF)

S2 Table Characteristics of included studies. (PDF)

S3 Table PRISMA Checklist. (PDF)
