# Correction: Communication Tools for End-of-Life Decision-Making in Ambulatory Care Settings: A Systematic Review and Meta-Analysis

**DOI:** 10.1371/journal.pone.0203911

**Published:** 2018-09-07

**Authors:** Simon J. Oczkowski, Han-Oh Chung, Louise Hanvey, Lawrence Mbuagbaw, John J. You

Fig 10 is incorrect. The authors have provided a corrected version here.

**Fig 10:**
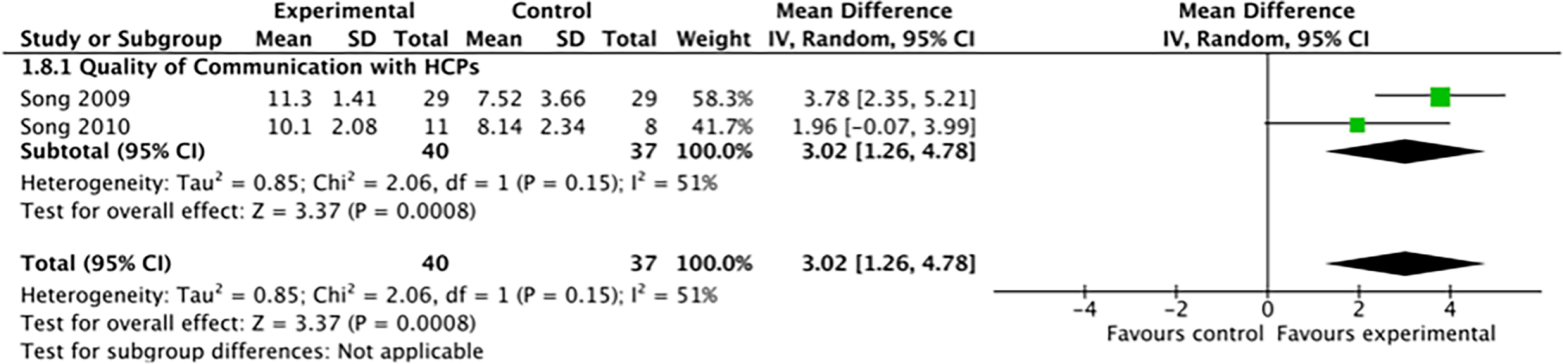
Quality of communication score between patients and health care providers.
