# Supplementary material for: Communication Tools for End-of-Life Decision-Making in Ambulatory Care Settings: A Systematic Review and Meta-Analysis
Source: PLoS One. 2016 Apr 27;11(4):e0150671. doi: 10.1371/journal.pone.0150671 (PMC4847908; doi:10.1371/journal.pone.0150671)

Supporting Information Figures: Sensitivity Analyses

Primary outcome 1a: Proportion of patients with documented ACP

	Communication tool		Usual care			Risk Ratio	
Study or Subgroup	Events	Total	Events	Total	Weight	M-H, Random, 95% CI	Year
**1.1.1 Low risk of bias studies**							
Molloy 2000	444	636	374	656	8.2%	1.22 [1.13, 1.33]	2000
Grimaldo 2001	63	99	57	99	7.7%	1.11 [0.88, 1.38]	2001
Heiman 2004	38	360	5	334	3.6%	7.05 [2.81, 17.70]	2004
Epstein 2013	19	30	15	26	6.5%	1.10 [0.72, 1.68]	2013
**Subtotal (95% CI)**		**1125**		**1115**	**26.1%**	**1.36 [0.98, 1.88]**	
Total events	564		451				
Heterogeneity: Tau² = 0.07; Chi² = 16.06, df = 3 (P = 0.001); I² = 81%							
Test for overall effect: Z = 1.86 (P = 0.06)							
**1.1.2 Unclear risk of bias studies**							
Rubin 1994	102	552	2	549	2.1%	50.72 [12.58, 204.55]	1994
Reilly 1995	11	83	9	79	4.1%	1.16 [0.51, 2.66]	1995
Landry 1997	33	95	20	92	6.2%	1.60 [0.99, 2.57]	1997
Dexter 1998	45	277	7	253	4.3%	5.87 [2.70, 12.78]	1998
Brown 1999	190	619	212	628	8.0%	0.91 [0.77, 1.07]	1999
Perry 2005	22	63	8	81	4.5%	3.54 [1.69, 7.40]	2005
Morrison 2005	17	43	19	96	5.7%	2.00 [1.16, 3.45]	2005
Pearlman 2005	57	136	30	144	6.8%	2.01 [1.38, 2.93]	2005
**Subtotal (95% CI)**		**1868**		**1922**	**41.7%**	**2.61 [1.40, 4.85]**	
Total events	477		307				
Heterogeneity: Tau² = 0.68; Chi² = 91.09, df = 7 (P < 0.00001); I² = 92%							
Test for overall effect: Z = 3.02 (P = 0.003)							
**1.1.3 High risk of bias studies**							
Sachs 1992	1	48	3	83	1.0%	0.58 [0.06, 5.39]	1992
High 1993	52	311	16	120	5.9%	1.25 [0.75, 2.11]	1993
Richter 1995	20	87	3	89	2.6%	6.82 [2.10, 22.13]	1995
Sulmasy 1996	0	63	1	101	0.5%	0.53 [0.02, 12.84]	1996
Yamada 1999	8	62	10	55	3.9%	0.71 [0.30, 1.67]	1999
Engelhardt 2006	60	133	48	142	7.3%	1.33 [0.99, 1.80]	2006
Sudore 2007	15	103	7	102	3.9%	2.12 [0.90, 4.99]	2007
Engelhardt 2009	78	267	41	265	7.1%	1.89 [1.35, 2.65]	2009
**Subtotal (95% CI)**		**1074**		**957**	**32.2%**	**1.53 [1.09, 2.15]**	
Total events	234		129				
Heterogeneity: Tau² = 0.09; Chi² = 14.07, df = 7 (P = 0.05); I² = 50%							
Test for overall effect: Z = 2.44 (P = 0.01)							
**Total (95% CI)**		**4067**		**3994**	**100.0%**	**1.77 [1.40, 2.23]**	
Total events	1275		887				
Heterogeneity: Tau² = 0.17; Chi² = 126.71, df = 19 (P < 0.00001); I² = 85%							
Test for overall effect: Z = 4.80 (P < 0.00001)							
Test for subgroup differences: Chi² = 3.29, df = 2 (P = 0.19), I² = 39.3%							

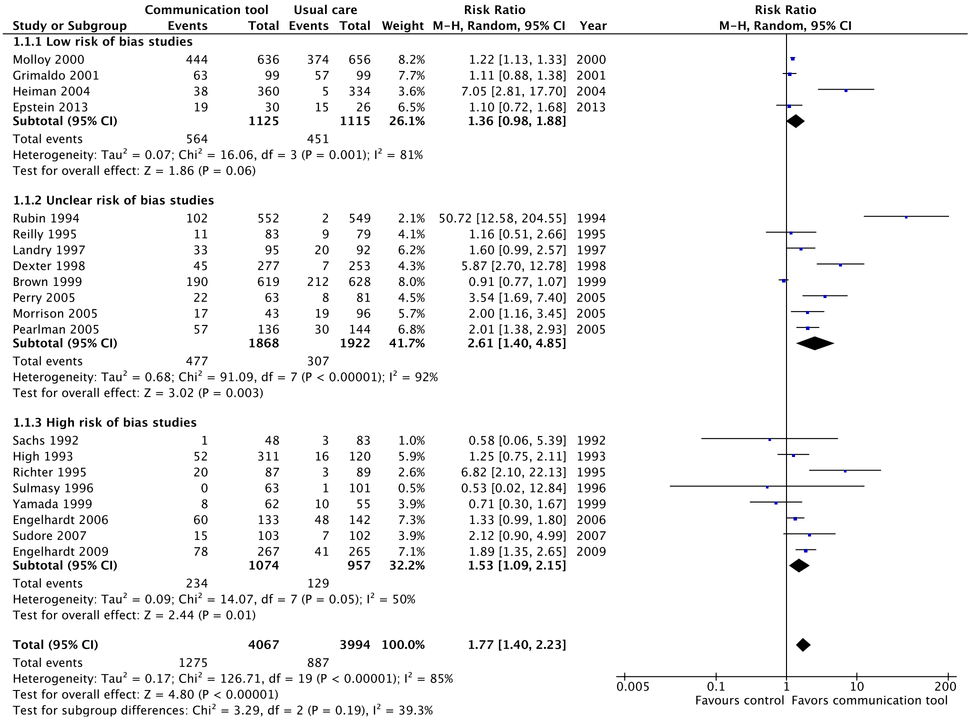

Primary outcome 1b: Documented ACP discussions

	Communication tool		Usual care			Risk Ratio	
Study or Subgroup	Events	Total	Events	Total	Weight	M-H, Random, 95% CI	Year
**1.2.1 Low risk of bias studies**							
Grimaldo 2001	31	99	25	99	14.8%	1.24 [0.79, 1.94]	2001
**Subtotal (95% CI)**		**99**		**99**	**14.8%**	**1.24 [0.79, 1.94]**	
Total events	31		25				
Heterogeneity: Not applicable							
Test for overall effect: Z = 0.94 (P = 0.35)							
**1.2.2 Unclear risk of bias studies**							
Dexter 1998	67	277	11	253	12.1%	5.56 [3.01, 10.28]	1998
Pearlman 2005	76	136	49	144	17.6%	1.64 [1.25, 2.16]	2005
Clayton 2007	27	92	8	82	10.4%	3.01 [1.45, 6.25]	2007
**Subtotal (95% CI)**		**505**		**479**	**40.1%**	**2.93 [1.25, 6.87]**	
Total events	170		68				
Heterogeneity: Tau² = 0.49; Chi² = 15.73, df = 2 (P = 0.0004); I² = 87%							
Test for overall effect: Z = 2.47 (P = 0.01)							
**1.2.3 High risk of bias studies**							
Sachs 1992	6	48	5	83	6.2%	2.08 [0.67, 6.44]	1992
Sulmasy 1996	15	86	10	101	10.1%	1.76 [0.83, 3.72]	1996
Heiman 2004	46	360	9	334	10.8%	4.74 [2.36, 9.54]	2004
Au 2012	117	194	56	182	18.0%	1.96 [1.53, 2.51]	2012
**Subtotal (95% CI)**		**688**		**700**	**45.2%**	**2.36 [1.51, 3.69]**	
Total events	184		80				
Heterogeneity: Tau² = 0.10; Chi² = 6.09, df = 3 (P = 0.11); I² = 51%							
Test for overall effect: Z = 3.76 (P = 0.0002)							
**Total (95% CI)**		**1292**		**1278**	**100.0%**	**2.30 [1.64, 3.23]**	
Total events	385		173				
Heterogeneity: Tau² = 0.15; Chi² = 26.08, df = 7 (P = 0.0005); I² = 73%							
Test for overall effect: Z = 4.80 (P < 0.00001)							
Test for subgroup differences: Chi² = 5.31, df = 2 (P = 0.07), I² = 62.3%							

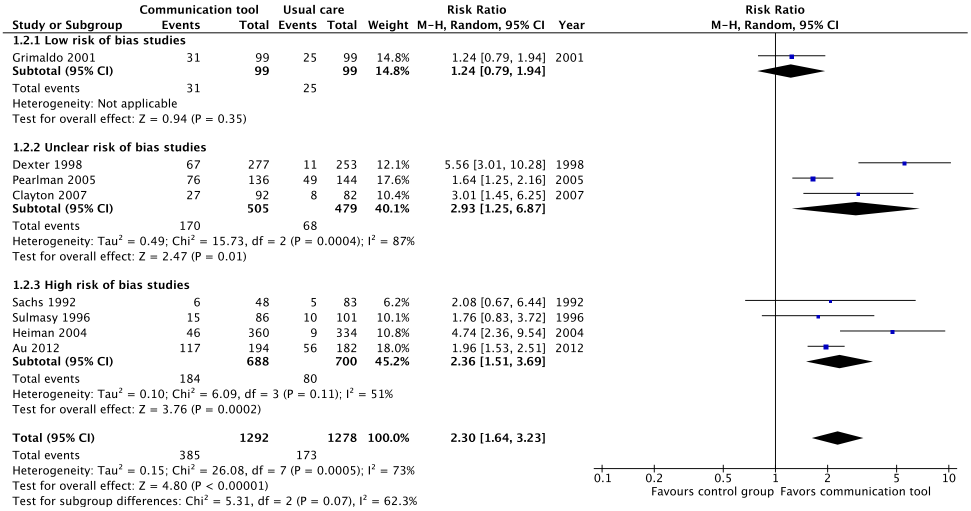

Secondary outcome 1: Patient preferences for life-prolonging as opposed to comfort care

Study or Subgroup	Communication tool Events	Communication tool Total	Usual care Events	Usual care Total	Weight	Risk Ratio M-H, Random, 95% CI
**1.13.1 Low risk of bias studies**						
Epstein 2013	5	30	4	26	2.1%	1.08 [0.32, 3.62]
Kirchhoff 2010	59	160	74	153	20.1%	0.76 [0.59, 0.99]
Rhondali 2013	10	45	9	35	4.5%	0.86 [0.39, 1.89]
Volandes 2009	3	94	6	106	1.7%	0.56 [0.15, 2.19]
Volandes 2011	0	33	7	44	0.4%	0.09 [0.01, 1.49]
Volandes 2012 c	6	50	18	51	4.0%	0.34 [0.15, 0.79]
**Subtotal (95% CI)**		**412**		**415**	**32.8%**	**0.67 [0.46, 0.97]**
Total events	83		118			
Heterogeneity: Tau² = 0.05; Chi² = 6.46, df = 5 (P = 0.26); I² = 23%						
Test for overall effect: Z = 2.13 (P = 0.03)						
**1.13.2 Unclear risk of bias studies**						
El-Jawahri 2010	1	23	7	27	0.8%	0.17 [0.02, 1.26]
**Subtotal (95% CI)**		**23**		**27**	**0.8%**	**0.17 [0.02, 1.26]**
Total events	1		7			
Heterogeneity: Not applicable						
Test for overall effect: Z = 1.73 (P = 0.08)						
**1.13.3 High risk of bias studies**						
Engelhardt 2009	183	267	195	265	31.1%	0.93 [0.84, 1.04]
Schneiderman 1992	34	104	40	100	14.2%	0.82 [0.57, 1.18]
Yamada 1999	41	62	39	55	21.1%	0.93 [0.73, 1.19]
**Subtotal (95% CI)**		**433**		**420**	**66.4%**	**0.92 [0.84, 1.02]**
Total events	258		274			
Heterogeneity: Tau² = 0.00; Chi² = 0.49, df = 2 (P = 0.78); I² = 0%						
Test for overall effect: Z = 1.63 (P = 0.10)						
**Total (95% CI)**		**868**		**862**	**100.0%**	**0.82 [0.68, 0.98]**
Total events	342		399			
Heterogeneity: Tau² = 0.02; Chi² = 15.18, df = 9 (P = 0.09); I² = 41%						
Test for overall effect: Z = 2.21 (P = 0.03)						
Test for subgroup differences: Chi² = 5.38, df = 2 (P = 0.07), I² = 62.8%						

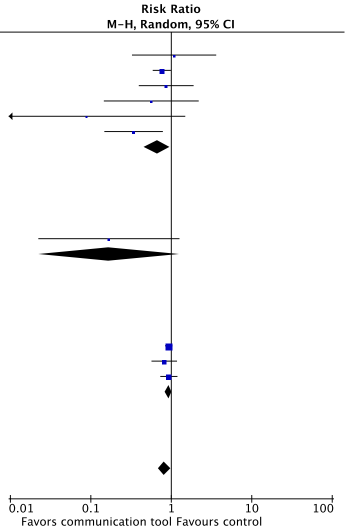

Secondary outcome 3: Quality of communication score between patient and health care provider

Study or Subgroup	Communication tool Mean	Communication tool SD	Communication tool Total	Usual care Mean	Usual care SD	Usual care Total	Weight	Std. Mean Difference IV, Random, 95% CI
**1.9.1 Low risk of bias studies**								
Song 2010	10.1	2.08	11	8.14	2.34	8	16.3%	0.85 [−0.11, 1.82]
**Subtotal (95% CI)**			**11**			**8**	**16.3%**	**0.85 [−0.11, 1.82]**
Heterogeneity: Not applicable								
Test for overall effect: Z = 1.74 (P = 0.08)								
**1.9.2 Unclear risk of bias studies**								
Song 2009	11.3	1.41	29	7.52	3.66	29	24.4%	1.34 [0.77, 1.92]
**Subtotal (95% CI)**			**29**			**29**	**24.4%**	**1.34 [0.77, 1.92]**
Heterogeneity: Not applicable								
Test for overall effect: Z = 4.59 (P < 0.00001)								
**1.9.3 High risk of bias studies**								
Au 2012	34	38.7	194	25.5	34.8	182	32.1%	0.23 [0.03, 0.43]
Jones 2011	4.1	6.6	43	3.7	9.91	34	27.3%	0.05 [−0.40, 0.50]
**Subtotal (95% CI)**			**237**			**216**	**59.4%**	**0.20 [0.01, 0.38]**
Heterogeneity: Tau² = 0.00; Chi² = 0.52, df = 1 (P = 0.47); I² = 0%								
Test for overall effect: Z = 2.11 (P = 0.03)								
**Total (95% CI)**			**277**			**253**	**100.0%**	**0.55 [0.01, 1.09]**
Heterogeneity: Tau² = 0.22; Chi² = 15.59, df = 3 (P = 0.001); I² = 81%								
Test for overall effect: Z = 2.01 (P = 0.04)								
Test for subgroup differences: Chi² = 15.07, df = 2 (P = 0.0005), I² = 86.7%								

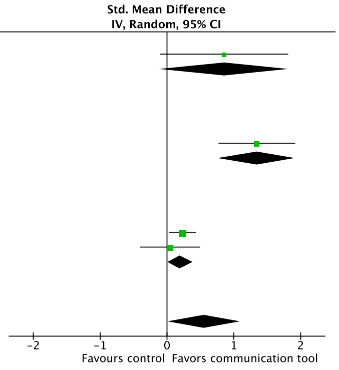

Secondary outcome 4a: Patient and family knowledge of life supporting treatments

	Communication tool			Usual care				Std. Mean Difference
Study or Subgroup	Mean	SD	Total	Mean	SD	Total	Weight	IV, Random, 95% CI
**1.6.1 Low risk of bias studies**								
Epstein 2013	4.9	2.1	30	4.9	2	26	14.9%	0.00 [−0.53, 0.53]
Volandes 2012	3.3	1	79	2.6	1.3	80	20.2%	0.60 [0.28, 0.92]
**Subtotal (95% CI)**			**109**			**106**	**35.0%**	**0.34 [−0.25, 0.92]**
Heterogeneity: Tau² = 0.13; Chi² = 3.67, df = 1 (P = 0.06); I² = 73%								
Test for overall effect: Z = 1.14 (P = 0.26)								
**1.6.2 Unclear risk of bias studies**								
Ditto 2001	4.75	0.45	82	4.36	0.57	80	20.1%	0.76 [0.44, 1.08]
El-Jawahri 2010	1.9	1.35	23	0.9	1.19	27	13.7%	0.78 [0.20, 1.36]
**Subtotal (95% CI)**			**105**			**107**	**33.8%**	**0.76 [0.48, 1.04]**
Heterogeneity: Tau² = 0.00; Chi² = 0.00, df = 1 (P = 0.95); I² = 0%								
Test for overall effect: Z = 5.34 (P < 0.00001)								
**1.6.3 High risk of bias studies**								
Dyar 2012	61.5	25.64	12	46.2	33.23	14	9.9%	0.49 [−0.29, 1.28]
Sudore 2007	71.2	15.6	103	70.8	14.9	102	21.3%	0.03 [−0.25, 0.30]
**Subtotal (95% CI)**			**115**			**116**	**31.1%**	**0.11 [−0.24, 0.46]**
Heterogeneity: Tau² = 0.02; Chi² = 1.22, df = 1 (P = 0.27); I² = 18%								
Test for overall effect: Z = 0.61 (P = 0.54)								
**Total (95% CI)**			**329**			**329**	**100.0%**	**0.43 [0.12, 0.75]**
Heterogeneity: Tau² = 0.10; Chi² = 17.40, df = 5 (P = 0.004); I² = 71%								
Test for overall effect: Z = 2.70 (P = 0.007)								
Test for subgroup differences: Chi² = 8.40, df = 2 (P = 0.01), I² = 76.2%								

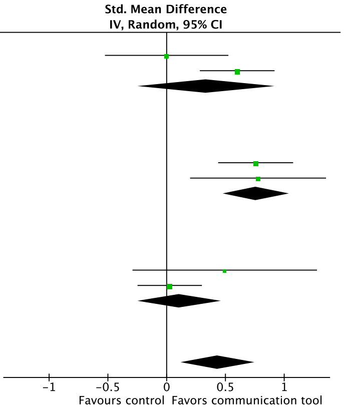

Secondary outcome 4b: Patient and family knowledge - advance care planning

	Communication tool			Usual care				Std. Mean Difference	
Study or Subgroup	Mean	SD	Total	Mean	SD	Total	Weight	IV, Random, 95% CI	Year
**1.7.1 Low risk of bias studies**									
Schwartz 2002	1.25	2.5	30	0.9	2.9	30	12.5%	0.13 [−0.38, 0.63]	2002
Kirchhoff 2010	8.26	2.1	160	7.52	2.15	153	64.3%	0.35 [0.12, 0.57]	2010
**Subtotal (95% CI)**			**190**			**183**	**76.8%**	**0.31 [0.11, 0.52]**	
Heterogeneity: Tau² = 0.00; Chi² = 0.61, df = 1 (P = 0.44); I² = 0%									
Test for overall effect: Z = 2.99 (P = 0.003)									
**1.7.2 Unclear risk of bias studies**									
Siegert 1996	7.5	3.6	16	6.9	2.4	20	7.4%	0.20 [−0.46, 0.86]	1996
Song 2005	8.38	1.36	16	7.75	2.14	16	6.6%	0.34 [−0.36, 1.04]	2005
**Subtotal (95% CI)**			**32**			**36**	**13.9%**	**0.27 [−0.21, 0.74]**	
Heterogeneity: Tau² = 0.00; Chi² = 0.09, df = 1 (P = 0.77); I² = 0%									
Test for overall effect: Z = 1.08 (P = 0.28)									
**1.7.3 High risk of bias studies**									
Gutheil 2005	5.3	1.6	27	3.82	1.9	22	9.2%	0.84 [0.25, 1.43]	2005
**Subtotal (95% CI)**			**27**			**22**	**9.2%**	**0.84 [0.25, 1.43]**	
Heterogeneity: Not applicable									
Test for overall effect: Z = 2.79 (P = 0.005)									
**Total (95% CI)**			**249**			**241**	**100.0%**	**0.35 [0.17, 0.53]**	
Heterogeneity: Tau² = 0.00; Chi² = 3.57, df = 4 (P = 0.47); I² = 0%									
Test for overall effect: Z = 3.87 (P = 0.0001)									
Test for subgroup differences: Chi² = 2.88, df = 2 (P = 0.24), I² = 30.5%									

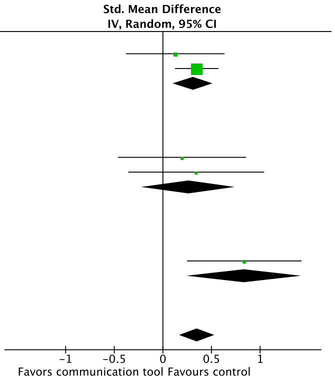

Secondary outcome 5: Patient and family satisfaction with end-of-life care

	Communication tool			Usual care				Std. Mean Difference	
Study or Subgroup	Mean	SD	Total	Mean	SD	Total	Weight	IV, Random, 95% CI	Year
**1.5.1 Low risk of bias studies**									
Clayton 2007	110.1	0.72	92	110.3	0.72	82	30.2%	−0.28 [−0.58, 0.02]	2007
**Subtotal (95% CI)**			**92**			**82**	**30.2%**	**−0.28 [−0.58, 0.02]**	
Heterogeneity: Not applicable									
Test for overall effect: Z = 1.81 (P = 0.07)									
**1.5.3 High risk of bias studies**									
Gutheil 2005	45.9	5.1	27	46	5.1	22	13.3%	−0.02 [−0.58, 0.54]	2005
Engelhardt 2006	4.07	0.68	133	3.98	0.67	142	37.1%	0.13 [−0.10, 0.37]	2006
Jones 2011	0.6	6.9	21	1.9	4.9	20	11.6%	−0.21 [−0.83, 0.40]	2011
Dyar 2012	2	25.73	12	−8.8	21.67	14	7.7%	0.44 [−0.34, 1.22]	2012
**Subtotal (95% CI)**			**193**			**198**	**69.8%**	**0.10 [−0.10, 0.30]**	
Heterogeneity: Tau² = 0.00; Chi² = 1.98, df = 3 (P = 0.58); I² = 0%									
Test for overall effect: Z = 0.96 (P = 0.33)									
**Total (95% CI)**			**285**			**280**	**100.0%**	**−0.03 [−0.26, 0.21]**	
Heterogeneity: Tau² = 0.02; Chi² = 6.15, df = 4 (P = 0.19); I² = 35%									
Test for overall effect: Z = 0.23 (P = 0.82)									
Test for subgroup differences: Chi² = 4.17, df = 1 (P = 0.04), I² = 76.0%									

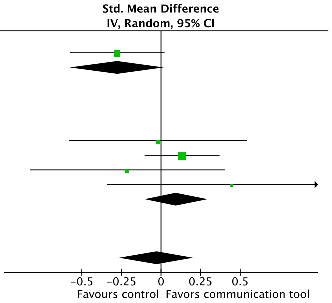

Supplement: S1 Fig — (PDF) [file pone.0150671.s001.pdf]
