# Supplementary material for: Communication Tools for End-of-Life Decision-Making in Ambulatory Care Settings: A Systematic Review and Meta-Analysis
Source: PLoS One. 2016 Apr 27;11(4):e0150671. doi: 10.1371/journal.pone.0150671 (PMC4847908; doi:10.1371/journal.pone.0150671)
Supplement: S1 Table — (PDF) [file pone.0150671.s002.pdf]

S1 Table: Electronic search strategies

Medline (1946-July 2014)

1. exp Hospital Communication Systems/ or exp Communication/ or exp Health Communication/ or exp Interdisciplinary Communication/ or exp Communication Barriers/ or exp Nonverbal Communication/
2. exp Physician-Patient Relations/ed, es, st [Education, Ethics, Standards]
3. *Family/ or exp Family Relations/
4. *Education, Medical, Undergraduate/ or *Clinical Competence/ or *Education, Medical, Graduate/
5. (communic$ adj3 tool$).mp. [mp=title, abstract, original title, name of substance word, subject heading word, keyword heading word, protocol supplementary concept, rare disease supplementary concept, unique identifier]
6. (communic$ adj3 skill$).mp. [mp=title, abstract, original title, name of substance word, subject heading word, keyword heading word, protocol supplementary concept, rare disease supplementary concept, unique identifier]
7. (communic$ adj3 intervention).mp. [mp=title, abstract, original title, name of substance word, subject heading word, keyword heading word, protocol supplementary concept, rare disease supplementary concept, unique identifier]
8. (education$ adj3 tool).mp. [mp=title, abstract, original title, name of substance word, subject heading word, keyword heading word, protocol supplementary concept, rare disease supplementary concept, unique identifier]
9. or/1-8
10. decision making/ or choice behavior/ or "dissent and disputes"/ or negotiating/
11. advance directives/ or living wills/ or contracts/ or informed consent/ or consent forms/ or third-party consent/ or presumed consent/ or resuscitation orders/
12. exp patient care planning/ or advance care planning/ or advance directives/ or living wills/ or critical pathways/ or exp patient-centered care/
13. (deci$ adj3 mak$).mp. [mp=title, abstract, original title, name of substance word, subject heading word, keyword heading word, protocol supplementary concept, rare disease supplementary concept, unique identifier]
14. advance care planning.mp.
15. or/10-14
16. exp Resuscitation/ or resuscitation.mp. or Cardiopulmonary Resuscitation/
17. exp life support care/ or advanced cardiac life support/ or advanced trauma life support care/
18. exp Terminal Care/ or exp Withholding Treatment/ or exp Life Support Care/ or withdrawing life support.mp. or Euthanasia, Passive/
19. Resuscitation Orders/ or dnr.mp.
20. critical illness/ or emergencies/
21. Emergency Service, Hospital/ or Hospital Rapid Response Team/
22. heart arrest.mp. or Heart Arrest/
23. Respiration, Artificial/ or artifical respiration.mp.
24. or/16-23
24. 9 and 15 and 24

Embase (1980-July 2014)

1. exp interpersonal communication/
2. family/ or family assessment/ or family decision making/ or family relation/
3. exp medical education/
4. exp doctor patient relation/
5. (communic$ adj3 tool$).mp.
6. (communic$ adj3 skill$).mp.
7. (communic$ adj3 intervention).mp.
8. (education$ adj3 tool).mp.
9. education program/ or educational intervention.mp.
10. or/1-9
11. exp medical decision making/ or decison making.mp. or exp decision making/
12. exp treatment planning/ or exp patient care planning/ or exp living will/ or care planning.mp. or exp decision making/
13. advance$ directives.mp.
14. advance$ care plan$.mp.
15. (deci$ adj3 mak$).mp.
16. or/11-15
17. exp resuscitation/ or exp emergency treatment/ or exp intensive care/
18. cardiopulmonary resuscitation.mp.
19. exp heart arrest/ or CPR.mp.
20. exp critical illness/
21. life support.mp.
22. Emergency Service, Hospital/ or Hospital Rapid Response Team/
23. Resuscitation Orders/ or dnr.mp.
24. Respiration, Artificial/ or artifical respiration.mp.
25. exp Terminal Care/ or exp Withholding Treatment/ or exp Life Support Care/ or withdrawing life support.mp. or Euthanasia, Passive/
26. or/17-25
27. 10 and 16 and 26

CINAHL (1982-July 2014)

Search ID#	Search Terms	Results
S1	(MH "Communication+") OR "communication" OR (MH "Communication Skills") OR (MH "Communication Skills Training") OR (MH "Communications Media+") OR (MH "Nonverbal Communication+") OR (MH "Communication Protocols+") OR (MH "Communication Barriers")	379,318
S2	(MH "Physician-Patient Relations") OR (MH "Professional-Patient Relations+") OR (MH "Nurse-Physician Relations")	51,346
S3	(MH "Family+") OR (MH "Family Relations+")	97,226
S4	(MH "Education, Medical+")	14,855
S5	S1 OR S2 OR S3 OR S4	496,220
S6	(MH "Decision Making+") OR (MH "Decision Making, Patient+") OR (MH "Decision Making, Family") OR (MH "Decision Making, Ethical") OR (MH "Decision Making, Clinical")	50,433
S7	(MH "Living Wills")	691
S8	(MH "Consent+")	10,526
S9	(MH "Resuscitation Orders")	1,731
S10	(MH "Advance Care Planning")	813
S11	(MH "Advance Directives+")	5,436
S12	S6 OR S7 OR S8 OR S9 OR S10 OR S11	62,104
S13	(MH "Resuscitation+") OR (MH "Resuscitation, Cardiopulmonary+") OR (MH "Bystander CPR")	19,882
S14	(MH "Advanced Cardiac Life Support+") OR (MH "Life Support Care")	2,425
S15	(MH "Terminal Care+") OR "terminal care" OR (MH "Palliative Care") OR (MH "Terminally Ill Patients+")	36,496
S16	(MH "Euthanasia, Passive") OR (MH "Treatment Refusal") OR (MH "Treatment Related Pain") OR (MH "Treatment Duration")	10,486
S17	(MH "Euthanasia, Passive")	2,748
S18	(MH "Heart Arrest+")	7,719
S19	(MH "Emergency Service+")	25,781
S20	(MH "Respiration, Artificial+") OR (MH "Positive Pressure Ventilation+") OR (MH "Ventilators, Mechanical")	13,909
S21	S13 OR S14 OR S15 OR S16 OR S17 OR S18 OR S19 OR S20	96,441
S22	S5 AND S12 AND S21	3,492
S23	S5 AND S12 AND S21	1,304

Cochrane Database of Clinical Controlled Trials (2005-July 2014)

1 (hospital communication systems or communication or health communication or interdisciplinary communication or communication barriers or nonverbal communication).mp. [mp=title, short title, abstract, full text, keywords, caption text] (1410)

2 Physician-Patient Relations.mp. [mp=title, short title, abstract, full text, keywords, caption text] (17)
3 (Education, Medical, Undergraduate or clinical competence or education, medical graduate).mp. [mp=title, short title, abstract, full text, keywords, caption text] (18)
4 (communic$ adj3 tool$).mp. [mp=title, short title, abstract, full text, keywords, caption text] (19)
5 (communic$ adj3 skill$).mp. [mp=title, short title, abstract, full text, keywords, caption text] (113)
6 (communic$ adj3 intervention).mp. [mp=title, short title, abstract, full text, keywords, caption text] (80)
7 (education$ adj3 tool).mp. [mp=title, short title, abstract, full text, keywords, caption text] (13)
8 (family or family relations).mp. [mp=title, short title, abstract, full text, keywords, caption text] (1736)
9 or/1-8 (2557)
10 (decision making or choice behavior or "dissent and disputes" or negotiating).mp. [mp=title, short title, abstract, full text, keywords, caption text] (802)
11 (advance directives or living wills or contracts or informed consent or consent forms or third-party consent or presumed consent or resuscitation orders).mp. [mp=title, short title, abstract, full text, keywords, caption text] (395)
12 (patient care planning or advance care planning or advance directives or living wills or critical pathways or patient-centered care).mp. [mp=title, short title, abstract, full text, keywords, caption text] (70)
13 (deci$ adj3 mak$).mp. [mp=title, short title, abstract, full text, keywords, caption text] (1041)
14 advance care planning.mp. [mp=title, short title, abstract, full text, keywords, caption text] (8)
15 or/10-14 (1374)
16 (resuscitation or Cardiopulmonary Resuscitation).mp. [mp=title, short title, abstract, full text, keywords, caption text] (245)
17 (life support care or advanced cardiac life support or advanced trauma life support care).mp. [mp=title, short title, abstract, full text, keywords, caption text] (11)
18 (Terminal Care or Withholding Treatment or Life Support Care or withdrawing life support or Euthanasia, Passive).mp. [mp=title, short title, abstract, full text, keywords, caption text] (52)
19 (resuscitation orders or DNR).mp. [mp=title, short title, abstract, full text, keywords, caption text] (4)
20 (critical illness or emergencies).mp. [mp=title, short title, abstract, full text, keywords, caption text] (154)
21 (Emergency Service, Hospital or Hospital Rapid Response Team).mp. [mp=title, short title, abstract, full text, keywords, caption text] (26)
22 Heart Arrest.mp. [mp=title, short title, abstract, full text, keywords, caption text] (18)
23 cardiac arrest.mp. [mp=title, short title, abstract, full text, keywords, caption text] (145)
24 (Respiration, Artificial or artifical respiration).mp. [mp=title, short title, abstract, full text, keywords, caption text] (87)
25 or/16-24 (611)
26 9 and 15 and 25 (61)

ERIC (1966-July 2014)

Search ID#	Search Terms
1	exp communication strategies/ or exp communications
2	exp Physician Patient Relationship/
3	(communic$ adj3 tool$).mp.
4	(communic$ adj3 skill$).mp.
5	(communic$ adj3 intervention$).mp.
6	(education$ adj3 tool$).mp.
7	exp Medical Education/
8	or/1-7
9	(medical decision making or decision making).mp. or exp decision making/
10	exp Medical Services/ or medical treatment.mp.
11	exp Death/ or exp Decision Making/ or advance directive$.mp.
12	living will.mp.
13	care planning.mp.
14	advance$ directive$.mp.
15	advance$ care plan$.mp.
16	(deci$ adj3 mak$).mp.
17	or/9-16
18	end-of-life.mp
19	(resuscitation or emergency or intensive or critical).mp.
20	exp first aid/ or CPR.mp. or cardiopulmonary.mp. or arrest.mp. or life support.mp.
21	DNR.mp.
22	or/18-21
23	8 and 17 and 22
