# Supplementary material for: Communication Tools for End-of-Life Decision-Making in Ambulatory Care Settings: A Systematic Review and Meta-Analysis
Source: PLoS One. 2016 Apr 27;11(4):e0150671. doi: 10.1371/journal.pone.0150671 (PMC4847908; doi:10.1371/journal.pone.0150671)
Supplement: S2 Table — (PDF) [file pone.0150671.s003.pdf]

Supporting information Table 2: Characteristics of included studies

Study ID	Sample Size	Population Description	Country	Design	Target of Intervention	Intervention	Comparator	Measured Outcome
Au 2012	Intervention 194<br>Control 182	Patients attending outpatient COPD clinics with outpatient responsible for their COPD care	USA	RCT (Cluster-randomized at level of HCP)	• Patient<br>• HCP	Patients completed baseline questionnaires, including preferences for advance directives. These were used to generate patient-specific feedback forms, which were shared with patients and their clinicians at their next scheduled visit.	Usual care	• Occurrence of EoL/AD Discussion<br>• Quality of Communication between SDM and Patient<br>• Quality of Communication with HCP
Barrio-Cantalejo 2009	Intervention 64<br>Control 59	Adults attending primary care clinics with an identified SDM	Spain	RCT (3 arms)	• Patient	Patients completed the regional advance directive form, plus had a shared discussion with their SDMs about goals of care.	Usual care	• Surrogate and Patient EoL Preference Congruence
Bonner 2014	Intervention 35<br>Control 35	SDMs of African-American patients with dementia, living in the community	USA	Pre/post	• Surrogate	ACT-Plan intervention: 4 once-weekly sessions to enhance knowledge, self-efficacy, and behaviours needed to make end-of-life treatment plans with dementia, guided by an advance nurse practitioner.	Four once-weekly sessions on health promotion topics.	• Knowledge/Literacy of EoL treatment options<br>• Preference on Life Sustaining Treatment Options<br>• Decisional Comfort/Confidence in EoL Plan
Briggs 2004	Intervention 13<br>Control 14	Patients age>50 recruited from congestive heart failure, dialysis, or cardiovascular surgery clinic, able to identify an SDM	USA	RCT	• Patient<br>• Surrogate	PC-ACP 1 hour meeting reviewing patient's medical conditions, advance care planning, patient values, and a summary.	Usual care	• Knowledge/Literacy of EoL treatment options<br>• Decisional Comfort/Confidence in EoL Plan<br>• Surrogate and Patient EoL Preference Congruence<br>• Quality of Communication with HCP
Brown 1999	Intervention 619<br>Control 628	Cognitively intact patients age>75 attending a primary care clinic	USA	RCT	• Patient	Intervention participants were sent the "You and Your Choices" plus the Peace of Mind: Advanced Directives Video, along with a follow-up postcard and questionnaire.	"You and Your Choices" written materials alone	• Advance Directives Completion<br>• Acceptability of Tool
Chan 2010	Intervention 59<br>Control 62	Cantonese-speaking adults age>65 with at least one moderate to severe health problem, living in a nursing home.	China	Cohort	• Patient	"Let Me Talk" advance care planning program elicits patient values and concerns, and preferences for end of life care, facilitated by a nurse practitioner, and summarized in a personalized booklet.	Usual care	• Occurrence of EoL/AD Discussion<br>• Knowledge/Literacy of EoL treatment options<br>• Quality of Communication between SDM and Patient
Clayton 2007	Intervention 92<br>Control 82	Patients with malignancy referred to palliative care services	Australia	RCT	• Patient	Palliative Care Question Prompt List (QPL), a 16 page booklet addressing 9 palliative care topics	Usual care	• Occurrence of EoL/AD Discussion<br>• Satisfaction with EoL care and care planning<br>• Acceptability of Tool
Deep 2010	120 Pre/Post participants	Patients age>40 attending an outpatient general medicine clinic, without close relationships to patients with dementia.	USA	Pre/post	• Patient	Verbal description of advanced dementia, followed by 2 minute video depicting a female patient with severe dementia, based on Functional Assessment Staging (FAST).	Verbal description alone	• Preference on Life Sustaining Treatment Options
Dexter 1998	Intervention 219<br>Control 253	Patients age>75 or age>50 and cardiac ischemia, CHF, chronic lung disease, cancer, cerebrovascular disease, CKD, cirrhosis	USA	RCT	• HCP	Physician EMR reminders to discuss ACP	Usual care	• Advance Directives Completion<br>• Occurrence of EoL/AD Discussion
Ditto 2001	Intervention 82<br>Control 80	Patients age >65 from outpatient primary care practices, with an identifiable SDM	USA	RCT	• Patient<br>• Surrogate	Valued Life Activities Directive, a value-based advance directive, followed by discussion with SDM about values and preferences under the supervision of interviewer	Usual care	• Knowledge/Literacy of EoL treatment options<br>• Decisional Comfort/Confidence in EoL Plan
Dyar 2012	Intervention 12<br>Control 14	Outpatient cancer clinic patients with metastatic cancer and expected referral to hospice within 12 months	USA	RCT	• Patient	"Five Wishes" and living will forms, and visit with an oncology nurse practitioner to address patient needs.	Usual care	• Satisfaction with EoL care and care planning<br>• Knowledge/Literacy of EoL treatment options
Einterz 2014	18 Pre/Post participants	Surrogate decision makers of patients with moderate to severe dementia living in a nursing home.	USA	Pre/post	• Surrogate	An 18 minute video decision aid on dementia prognosis, treatment, elicitation of values and principles of substitute decision-making for end-of-life decisions, followed by a structured meeting with the SDM, patient, and interdisciplinary team within 3 months.	N/A	• Satisfaction with EoL care and care planning<br>• Knowledge/Literacy of EoL treatment options<br>• Quality of Communication with HCP<br>• Surrogate and HCP EoL Goals of care congruence
El-Jawahri 2010	Intervention 23<br>Control 27	Oncology clinic patients with a diagnosis of malignant glioma.	USA	RCT	• Patient	Brief (6 minute) video presenting three levels of medical care: life-prolonging (ICU, ventilation, CPR); basic medical care (IV medications and oxygen); and comfort care (hospice with medications and help with self-care	Verbal description of the same three levels of medical care.	• Knowledge/Literacy of EoL treatment options<br>• Preference on Life Sustaining Treatment Options<br>• Decisional Comfort/Confidence in EoL Plan<br>• Acceptability of Tool
Engelhardt 2006	Intervention 133<br>Control 142	Outpatients at high risk of death due to cancer, CHF, or COPD, from clinics at 3 veteran's centres, a home care organization, and two managed care organizations	USA	RCT	• Patient	Advanced illness coordinated care program, a six-session in intervention with counselling, education, and care coordination	Usual care	• Advance Directives Completion<br>• Health Care Utilization<br>• Satisfaction with EoL care and care planning
Engelhardt 2009	Intervention 267<br>Control 265	Outpatients at high risk of death due to cancer, CHF, pulmonary disease, or ESRD in one health care system	USA	RCT	• Patient	Advanced illness coordinated care program, a six-session in intervention with counselling, education, and care coordination	Usual care	• Advance Directives Completion<br>• Health Care Utilization
Epstein 2013	Intervention 30<br>Control 26	Outpatients with advanced, incurable GI malignancies from a regional oncology clinic who had not completed advanced directives	USA	RCT	• Patient	A 3 minute video with narration and images of CPR and mechanical ventilation, and probability of survival for patients with advanced cancer	Identical information presented orally, without the video	• Advance Directives Completion<br>• Health Care Utilization<br>• Advance Directives Completion<br>• Knowledge/Literacy of EoL treatment options<br>• Preference on Life Sustaining Treatment Options<br>• Acceptability of Tool
Fung 2010	100 Pre/Post participants	Participants recruited from residential homes and seniors community centers; capable of understanding Cantonese	China	Pre/post	• Patient	Two hypothetical scenarios, one about dementia and the other about COPD,were used to discuss decision-making with regard to life-sustaining therapies.	N/A	• Knowledge/Literacy of EoL treatment options
Grimaldo 2001	Intervention 99<br>Control 99	Patients age > 65 scheduled for surgery anticipated to require hospital admission overnight	USA	RCT	• Patient	10 minute information session discussing CPR, mechanical ventilation, and the importance of discussing EOL with their SDM. All patients received an advanced directives form.	Usual care	• Advance Directives Completion<br>• Occurrence of EoL/AD Discussion<br>• Preference on Life Sustaining Treatment Options<br>• Quality of Communication between SDM and Patient
Gutheil 2005	Intervention 27<br>Control 22	Elderly patients enrolled in a seniors centre with an identified substitute decision maker.	USA	RCT	• Patient<br>• Surrogate	Four 1-hour teaching sessions on advance directives, substitute decision making, communication of values, and review of advance directives forms.	Usual care	• Satisfaction with EoL care and care planning<br>• Knowledge/Literacy of EoL treatment options<br>• Quality of Communication between SDM and Patient
Hanson 2005	346 Pre/Post participants	Residents of seven community nursing homes	USA	Pre/post	• Surrogate	Quality improvement program in palliative care, including creation of a palliative care team with skills in advance care planning, and creation of procedures and documentation tools to enhance ACP.	Usual care	• Advance Directives Completion<br>• Occurrence of EoL/AD Discussion<br>• Concordance documented AD and desired care
Hare 1991	Intervention 52<br>Control 55	Adult patients from a tertiary-care outpatient internal medicine clinic.	USA	Cohort	• Patient	Patients received a booklet as well as a physician-initiated advance directives discussion at their scheduled clinic visit.	Usual care	• Advance Directives Completion
Heffner 1997	Intervention 50<br>Control 43	Adult patients attending outpatient pulmonary rehabilitation	USA	Cohort	• Patient	Educational workshop on advanced directives and end-of-life issues, including a 15 minute video and discussion led by a nurse educator. Patients were given AD forms to complete.	Usual care	• Advance Directives Completion<br>• Occurrence of EoL/AD Discussion<br>• Quality of Communication with HCP
Heiman 2004	Intervention 385<br>Control 334	Patients age>70 or age>50 with HIV, cancer, cardiomyopathy, pulmonary edema, CVA, cirrhosis, ESRD, CKD, COPD, paraplegia or quadriplegia, ALS	USA	RCT (Cluster)	• HCP	Physician EMR reminder to discuss ACP	Usual care	• Advance Directives Completion<br>• Occurrence of EoL/AD Discussion
High 1993	Intervention 311<br>Control 120	Patients recruited from 8 seniors homes, a volunteer pool of elderly at the University, and two geriatric clinics	USA	RCT	• Patient	Written materials plus meeting	Meetings alone	• Advance Directives Completion
Hossler 2011	17 Pre/Post participants	Individuals being treated at a clinic for amyotrophic lateral sclerosis	USA	Pre/post	• Patient	Participants completed a computer-based decision aid to clarify values and make advance care plans	N/A	• Decisional Comfort/Confidence in EoL Plan<br>• Acceptability of Tool
Jones 2011	Intervention 43<br>Control 34	Oncology patients who had completed a course of treatment for malignancy with residual active disease.	UK	RCT	• Patient	Meeting with trained facilitator with checklist to discuss end-of-life issues. Patients provided with ADs to complete.	Usual care	• Satisfaction with EoL care and care planning<br>• Quality of Communication between SDM and Patient<br>• Quality of Communication with HCP
Kirchoff 2010	Intervention 160<br>Control 153	Adult outpatients from congestive heart failure or dialysis clinics	USA	RCT	• Patient<br>• Surrogate	"Respecting Choices" intervention. Participants received to pamphlets which describe ACP and prompt them to consider personal goals and values for end of life care, followed by a discussion with a trained facilitator, and documentation.	Usual care	• Knowledge/Literacy of EoL treatment options<br>• Quality of Communication with HCP
Kirchoff 2012	Intervention 160<br>Control 153	Adult patients with end-stage renal disease or congestive heart failure at two hospital clinics	USA	RCT	• Patient<br>• Surrogate	Patients with SDMs were interviewed by a skilled facilitator to assess understanding of disease, treatment options, and documentation of patient preferences	Usual care	• Concordance of desired care and delivered care<br>• Preference on Life Sustaining Treatment Options
Kressel 2007	Intervention 39<br>Control 36	Patients age>65 attending an academic general internal medicine practice	USA	Cohort	• Patient	Patients in the intervention arm reviewed an advanced directive where the check box allowed one to 'opt out' of treatment	Patients in the control reviewed an advance directive where the check box allowed one to 'opt in' to treatments	• Preference on Life Sustaining Treatment Options
Landry 1997	Intervention 95<br>Control 92	Adult patients from the general internal medicine clinic	USA	RCT	• Patient	Educational seminar- 10 minute presentation on advance directives, organ donation, power of attourney, 15 minute presentation on advance directive, 30 minute discussion period, 10 minutes to fill out advance directives form	Information packet and advance directive form	• Advance Directives Completion<br>• Quality of Communication between SDM and Patient
Molloy 2000	Intervention 636<br>Control 656	6 matched nursing homes for elderly patients	Canada	RCT (Cluster)	• Patient	Let me decide' advance directive along with meeting with health care facilitators to discuss treatment options	Usual care	• Advance Directives Completion
Morrison 2005	Intervention 43<br>Control 96	Adults admitted to a long-term using home	USA	RCT	• HCP	Social workers received half day training on conducting ACP discussions, capacity asesssment	Usual care	• Advance Directives Completion<br>• Concordance of desired care and delivered care
Murphy 1994	287 Pre/Post participants	Adults age>60 attending a ambulatory geriatric clinic	USA	Pre/post	• Patient	Patients were shown a pie chart of CPR outcomes, including survival and functional status	N/A	• Preference on Life Sustaining Treatment Options
Murphy 2000	31 Pre/Post participants	Senior citizens attending a multipurpose community senior citizens centre	USA	Pre/post	• Patient	Computer program reviewing information on advance directives, treatment options, and how to express preferences and values.	N/A	• Satisfaction with EoL care and care planning<br>• Knowledge/Literacy of EoL treatment options
Pearlman 2005	Intervention 136<br>Control 144	Patients aged >55 recruited from from primary care clinics	USA	RCT	• Patient<br>• HCP	Mailed "Your Life, Your Choices" booklet, a 30 minute appointment with a social worker, and an appointment flag with the care provider to discuss ACP	Usual care plus the hospital's standard advance directives package	• Advance Directives Completion<br>• Occurrence of EoL/AD Discussion<br>• Surrogate and Patient EoL Preference Congruence
Perry 2005	Intervention 63<br>Control 59	Outpatients from 21 dialysis units who had not completed advanced directives	USA	RCT	• Patient	Peer mentoring from ERSD patients trained to discuss AD; 5 telephone meetings and 3 face-to-face, for the purpose of education about AD and addressing worries about AD	Printed materials on advanced directives	• Advance Directives Completion
Reilly 1995	Intervention 83<br>Control 79	Patients recently discharged from hospital admission to home, without prior advance directives	USA	RCT	• Patient	Inteviewed by physician during admission regarding prior advance care plans, mailed 8-page advanced directives booklet including advance directives forms	Usual care, including interview during admission by physician regarding advance care planning	• Advance Directives Completion
Reinke 2011	7 Pre/Post participants	Patients with severe COPD, previous participation in dyspnea self-management program, and were interested in learning about end-of-life issues.	USA	Pre/post	• Patient	60 minute webinar and online open peer-group discussions on end-of-life topics and decision-making.	N/A	• Satisfaction with EoL care and care planning<br>• Acceptability of Tool<br>• Quality of Communication between SDM and Patient
Rhondali 2013	Intervntion 45<br>Control 35	Patients with advanced cancer attending a supportive care clinic	USA	RCT (Crossover)	• Patient	Video of physician asking what the patient preferred in the event of cardiac arrest, crossing over to a video of the physician recommending DNR	Video of the physician recommending DNR, followed by a video of the physician asking what the patient preferred in the event of cardiac arrest	• Satisfaction with EoL care and care planning<br>• Preference on Life Sustaining Treatment Options
Richter 1995	Intervention 87<br>Control 89	Adult patients at an internal medicine outpatient clinic	USA	RCT	• Patient	Patients were given advanced directives forms and information materials at their clinic visit, with a mailed reminder 2 weeks later, with a request to have the forms completed by a certain date.	Usual care, with a copy of an advanced directives brochure from the clinic nurse.	• Advance Directives Completion
Rubin 1994	Intervention 552<br>Control 549	Patients age>65 recently discharged from a hospital stay of 3 days or greater	USA	RCT	• Patient	Patients were mailed a letter, a form reviewing advance directives and a power of attorney form, with follow-up letter and forms sent again at 4 week follow-up	Usual care	• Advance Directives Completion
Sachs 1992	Intervention 48<br>Control 83	Patients age>65 with more than one visit to a university geriatrics clinic	USA	RCT	• Patient	30 minute interview exploring knowledge of advance directives, and teaching involving case vignettes. Patients were give advance directive forms, and a reminder card to discuss AD at next clinic visit.	Usual care	• Advance Directives Completion<br>• Occurrence of EoL/AD Discussion
Schneiderman 1992	Intervention 104<br>Control 100	Patients with life threatening disease, with estimated life expectancy <5 years	USA	RCT	• Patient	Discussion about advance directives in clinic, and provision of advance directives forms	Usual care	• Preference on Life Sustaining Treatment Options<br>• Health Care Utilization
Schonwetter 1991	64 Pre/Post participants	Patients aged 75 or greater from an outpatient geriatrics patient	USA	Pre/post	• Patient	Patients were given a description of cardiac arrest, CPR, risks and benefits of treatment, and told that the decision whether or not to undergo CPR was up to the patient.	N/A	• Knowledge/Literacy of EoL treatment options
Schonwetter 1993	102 Pre/Post participants	Adults residing in an retirement-home community	USA	Pre/post	• Patient	Patients were given a description of cardiac arrest, CPR, risks and benefits of treatment, and provide CPR preferences for 5 scenarios.	N/A	• Knowledge/Literacy of EoL treatment options
Schwartz 2002	Intervention 30<br>Control 30	Ambulatory geriatric patients with a chronic or life-threatening disease.	USA	RCT	• Patient	"Respecting Choices" intervention. Participants received to pamphlets which describe ACP and prompt them to consider personal goals and values for end of life care, followed by a discussion with a trained facilitator, and documentation.	Local advance directives forms to take home and complete.	• Knowledge/Literacy of EoL treatment options<br>• Surrogate and Patient EoL Preference Congruence
Siegert 1996	Intervention 16<br>Control 20	Patients age greater than 40 staying in an extended care and rehabilitation centre	USA	RCT	• Patient	Participants received written educational materials on advance directives, followed by a video on advance directives.	Written educational materials, followed by a video tape on diet and exercise	• Knowledge/Literacy of EoL treatment options
Singer 1995	Intervention 95<br>Control 95	Patients recruited from 6 dialysis units in Toronto	Canada	RCT	• Patient	Dialysis-specific advance directive	Generic advance directive form	• Preference for completing advance directive<br>• Decisional Comfort/Confidence in EoL Plan<br>• Acceptability of Tool
Singer 1997	Intervention 101<br>Control 101	Patients with HIV recruited from AIDS committee of Toronto and the hospital HIV clinic	Canada	RCT	• Patient	HIV-specific advance directive	Generic advance directive form	• Preference for completing advance directive<br>• Decisional Comfort/Confidence in EoL Plan<br>• Acceptability of Tool
Smucker 1993	Intervention 85<br>Control 15	Patients age>65 attending an urban family preactice clinic	USA	RCT	• Patient	Physician initiated discussion on advance directives	Usual care	• Surrogate and HCP EoL Goals of care congruence
Song 2005	Intervention 16<br>Control 16	Patients age>50 in cardiac surgery clinic, scheduled for an operating, with an identified SDM	USA	RCT	• Patient<br>• Surrogate	PC-ACP 1 hour meeting reviewing patient's medical conditions, advance care planning, patient values, and a summary.	Usual care	• Knowledge/Literacy of EoL treatment options<br>• Decisional Comfort/Confidence in EoL Plan<br>• Surrogate and Patient EoL Preference Congruence
Song 2009	Intervention 29<br>Control 29	Self-identified African-americans receiving dialysis for at least 3 months, with an identified SDM	USA	RCT	• Patient	SPIRIT, a one hour interview with a patient and SDM by a trained nurse practitioner, to explore values, beliefs, and advance directives.	Social worker at the dialysis clinic provided written information on advance directives.	• Quality of Communication with HCP
Song 2010	Intervention 11<br>Control 8	African-american patients receiving hemodialysis for 3 or more months, with an identified SDM	USA	RCT	• Patient<br>• Surrogate	PC-ACP 1 hour meeting reviewing ESRD, advance care planning, patient values, and a summary, in addition to written information on advance directives provided by nurse or social worker.	Written information on advance directives provided by nurse or social worker.	• Surrogate and Patient EoL Preference Congruence<br>• Quality of Communication with HCP
Sudore 2007	Intervention 103<br>Control 102	Cognitively intact patients age>50, attending a general medicine clinic	USA	RCT	• Patient	Intervention patients received a revised advance directives form with large font and simplified text at a 5th grade reading level.	Standard advance directives form	• Advance Directives Completion<br>• Knowledge/Literacy of EoL treatment options<br>• Acceptability of Tool
Sudore 2014	43 Pre/Post participants	English speaking adults over age 60 from three low-income seniors centers	USA	Pre/post	• Patient	PREPARE: a web-based program to help patients with ACP, and selection and communication of ACP to the SDM and physicians.	N/A	• Advance Directives Completion<br>• Occurrence of EoL/AD Discussion<br>• Quality of Communication between SDM and Patient
Sulmasy 1996	Intervention 63<br>Control 101	Patients>18 and <90 who had twice visited an internal medicine ambulatory care practice within the preceding 3 months	USA	RCT	• Patient	Patients received a booklet about advance directives and a copy of advance directive forms to discuss with their physician on that visit. Follow up visit with a graduate student before or after the visit were also offered	Usual care	• Advance Directives Completion<br>• Occurrence of EoL/AD Discussion
Volandes 2007	120 Pre/Post participants	Patients age>40 attending an outpatient general medicine clinic, without close relationships to patients with dementia.	USA	Pre/post	• Patient	Verbal description of advanced dementia, followed by a 2 video depicting advanced dementia.	N/A	• Preference on Life Sustaining Treatment Options
Volandes 2009 (a)	Intervention 94<br>Control 106	Adults age >65 from outpatient geriatric and primary care clinics	USA	RCT	• Patient	Verbal description of advanced dementia, followed by video decision support tool showing advanced dementia in a nursing home setting.	Verbal description alone	• Preference on Life Sustaining Treatment Options
Volandes 2009 (b)	Intervention 8<br>Control 6	Patients age>65, with identified SDMs, attending outpatient geriatric clinics	USA	RCT	• Patient<br>• Surrogate	Verbal description of advanced dementia, followed by a 2 video depicting advanced dementia.	Written description alone	• Surrogate and Patient EoL Preference Congruence
Volandes 2010	146 Pre/Post participants	Patients age>40 attending an outpatient general medicine clinic, without close relationships to patients with dementia.	USA	Pre/post	• Patient	Verbal description of advanced dementia, followed by a 2 video depicting advanced dementia.	Verbal description alone	• Preference on Life Sustaining Treatment Options<br>• Decisional Comfort/Confidence in EoL Plan
Volandes 2011	33 Intervention<br>44 Control	Patients age>65 attending an outpatient primary care clinic	USA	RCT	• Patient	Patients received a verbal description of advanced dementia, followed by a video showing three levels of care: life prolonging (CPR, MV, ICU); basic care (hospital, IV, oxygen) and comfort (medications, hospice) followed by a 6 minute video illustrating the three choices.	Verbal description of dementia and different levels of care alone	• Preference on Life Sustaining Treatment Options
Volandes 2012 (a)	Intervention 79<br>Control 80	Oncology cancer patients with life expectancy less than 1 year.	USA	RCT	• Patient	Verbal narrative about CPR followed by 3 minute video demonstrating chest compressions, intubation, and a ventilated patient receiving care in ICU	Verbal narrative of CPR alone	• Knowledge/Literacy of EoL treatment options<br>• Preference on Life Sustaining Treatment Options
Volandes 2012 (b)	80 Pre/Post participants	Patients with advanced cancer, whose primary goals of treatment were palliative, from an outpatient oncology centre.	USA	Pre/post	• Patient	Patients received a verbal description of three levels of care: life prolonging (CPR, MV, ICU); basic care (hospital, IV, oxygen) and comfort (medications, hospice) followed by a 6 minute video illustrating the three choices.	N/A	• Preference on Life Sustaining Treatment Options
Volandes 2012 (c)	Intervention 50<br>Control 51	Patients age >65 residing in a skilled nursing facility	USA	RCT	• Patient	Brief (6 minute) video presenting three levels of medical care: life-prolonging (ICU, ventilation, CPR); basic medical care (IV medications and oxygen); and comfort care (hospice with medications and help with self-care	Verbal description of three levels of care alone	• Preference on Life Sustaining Treatment Options<br>• Acceptability of Tool
Wilson 2005	33 Pre/Post participants	Patients with severe COPD attending an outpatient pulmonary rehab unit.	Canada	Pre/post	• Patient	Participants received a portable, self-administered audio booklet, describing the prognosis of COPD, and the risks and benefits of mechanical ventilation.	N/A	• Preference on Life Sustaining Treatment Options<br>• Decisional Comfort/Confidence in EoL Plan
Wissow 2004	Intervention 842<br>Control 1278	Elderly patients (age>65) attending ambulatory clinics in a major health system	USA	Cohort	• HCP<br>• System	Multiple interventions to improve use of AD, including a presentation to MDs, site visits, booklet with information on ACP	Usual care	• Advance Directives Completion
Yamada 1999	Intervention 62<br>Control 55	Veterans age>70, deemed capable of medical decision-making	USA	RCT	• Patient	Pamphlet about CPR and outcomes, 10 minute video about advanced directives, and pamphlet about advanced directives that does not discuss CPR	Pamphlet about advanced directives which does not does not discuss CPR	• Advance Directives Completion<br>• Knowledge/Literacy of EoL treatment options<br>• Preference on Life Sustaining Treatment Options<br>• Quality of Communication between SDM and Patient
